# Model-based analysis of N-glycosylation in Chinese hamster ovary cells

**DOI:** 10.1371/journal.pone.0175376

**Published:** 2017-05-09

**Authors:** Frederick J. Krambeck, Sandra V. Bennun, Mikael R. Andersen, Michael J. Betenbaugh

**Affiliations:** 1 Department of Chemical and Biomolecular Engineering, Johns Hopkins University, Baltimore, Maryland, United States of America; 2 ReacTech Inc., Alexandria, Virginia, United States of America; 3 Department of Systems Biology, Technical University of Denmark, Lyngby, Denmark; Swiss Institute of Bioinformatics, SWITZERLAND

## Abstract

The Chinese hamster ovary (CHO) cell is the gold standard for manufacturing of glycosylated recombinant proteins for production of biotherapeutics. The similarity of its glycosylation patterns to the human versions enable the products of this cell line favorable pharmacokinetic properties and lower likelihood of causing immunogenic responses. Because glycan structures are the product of the concerted action of intracellular enzymes, it is difficult to predict a priori how the effects of genetic manipulations alter glycan structures of cells and therapeutic properties. For that reason, quantitative models able to predict glycosylation have emerged as promising tools to deal with the complexity of glycosylation processing. For example, an earlier version of the same model used in this study was used by others to successfully predict changes in enzyme activities that could produce a desired change in glycan structure. In this study we utilize an updated version of this model to provide a comprehensive analysis of N-glycosylation in ten Chinese hamster ovary (CHO) cell lines that include a wild type parent and nine mutants of CHO, through interpretation of previously published mass spectrometry data. The updated N-glycosylation mathematical model contains up to 50,605 glycan structures. Adjusting the enzyme activities in this model to match N-glycan mass spectra produces detailed predictions of the glycosylation process, enzyme activity profiles and complete glycosylation profiles of each of the cell lines. These profiles are consistent with biochemical and genetic data reported previously. The model-based results also predict glycosylation features of the cell lines not previously published, indicating more complex changes in glycosylation enzyme activities than just those resulting directly from gene mutations. The model predicts that the CHO cell lines possess regulatory mechanisms that allow them to adjust glycosylation enzyme activities to mitigate side effects of the primary loss or gain of glycosylation function known to exist in these mutant cell lines. Quantitative models of CHO cell glycosylation have the potential for predicting how glycoengineering manipulations might affect glycoform distributions to improve the therapeutic performance of glycoprotein products.

## Introduction

Many commercial proteins that are critical for treating diseases contain oligosaccharides that influence their functions, properties and yield. For that reason, biomanufacturers are focused on controlling the glycoform distribution of their biotherapeutics. N–glycosylation takes place through the action of a complex sequence of enzyme-catalyzed reactions that add or remove sugars to the glycan chains and generate a wide diversity of glycan structures [1,2,3,4].

The final goal to optimize glycosylation for therapeutic applications is to mimic human type glycosylation. For that end, mammalian cells are currently employed because of their similar glycoform distributions to human cells, with the Chinese hamster ovary (CHO) cell being the major mammalian cell platform for the industrial production of glycosylated biotherapeutics [5].

Mutants of CHO cells have been in particular important for metabolic oligosaccharide engineering of recombinant proteins [6,7,8,9,10].Indeed, the glycoforms of pharmaceutical proteins obtained from diverse cell lines have been extensively examined and been determined to have profound effects on the efficacy of glycoprotein pharmaceuticals. Examples include the presence/absence of proximal α-1,6-linked fucose, which can affect the efficacy of the biopharmaceutical as much as 100-fold [11], and the extent of terminal sialylation affecting serum half-life [12]. Expression of GnTIII led to increase of the antibody-dependent cell-mediated cytotoxicity (ADCC)ofchCE7 monoclonal antibodies (mAbs) [13]. Various methods have been employed to affect glycan structures from genetic manipulations [14,15,8,16,10] to variations in the cell culture processing parameters [17,18].

However, in mammalian expression platforms it is difficult to predict how the network of thousands of enzyme-catalyzed reactions interact to produce the great diversity of glycan structures. Complicating factors include the competitive action of multiple enzymes on each substrate and multiple substrates on each enzyme and the localization of the enzymes to specific Golgi compartments.

In order to gain predictive power of glycan modifications, structured models of the glycosylation processes have emerged as a complementary approach. Significant progress has already been made in the development of CHO glycosylation models. Umana and Bailey predicted 33 N-glycan structures using a model with 8 enzymes in 4 compartments, modeled as well-mixed reactors in series, and limited to reactions up to the first galactosylation steps [19]. Values of the model parameters were estimated using literature data, emphasizing CHO cells. The predicted glycans were similar to the experimental glycan distributions for recombinant proteins produced in CHO cells (tPA, EPO, β-interferon) [19]. Based on the Umana and Bailey model, Krambeck and Betenbaugh [4] developed an extended N-glycosylation model (KB2005) that went beyond the first galactosylation step and included additional enzymes for fucosylation, formation of N-acetyllactosamine repeats and sialylation, in total 11 enzymes predicting 7565 glycans. Lactosamine groups were limited to two per branch to limit the size of the model. Again, initial estimates of all the model parameters were based on literature data. The model predictions of major glycosylation profile features for recombinant human thrombopoietin (rhTPO) in CHO cells were brought into quantitative agreement with the experimental data from Inoue et al. [20] by adjusting the overall model enzyme concentrations and adding an adjustment rule for one enzyme without changing any other model parameters.

Essentially the same KB2005 model, with just the GnTIII enzyme removed (3677 components and 11,541 reactions), was more recently used by McDonald et al.[21] to predict what changes in enzyme activities would be required to increase N-glycan branching. In addition tithe obvious increases in GnTIV and GnTV enzyme activities, the model predicted that a decrease in galactosyltransferase activity would significantly increase the desired structures. They then glycoengineered a number of cell lines with various shifts in enzyme expression and verified this prediction experimentally. The original model with 7565 components was also used by St. Armand et al.[22] to analyze the controllability of N-glycosylation in CHO cells.

Hossler et al. [23] modeled the Golgi network as four plug-flow reactors in series based on the Golgi maturation theory of Golgi network transport, as opposed to the vesicular transport theory that motivated the four well-mixed reactor model. Comparing the plug-flow approximation to the well-mixed approximation, the authors concluded that the plug-flow approximation was probably more accurate. This model could also be considered a single plug-flow reactor with the enzymes distributed along its length in "box-car" fashion.

Jimenez del Val et al. [24] more recently pursued the Golgi maturation concept further, representing the Golgi apparatus by a single plug-flow reactor with the various enzymes located along its length in Gaussian-shaped distributions. This model also includes nucleotide-sugar transporter proteins, also located in Gaussian distributions along the reactor length. The enzyme and transporter distributions were based on an optimization procedure minimizing the total amount of enzyme or transporter required. Eight enzymes were included to generate 77 structures and 95 reactions. The authors achieved good agreement to a variety of experimental results for N-glycosylation of monoclonal antibodies. They also compared their model to their own implementations of both the KB2005 and Hossler models using their reaction network. The authors found that their model did a significantly better job of matching the data than the other two when only the total enzyme concentrations were adjusted for the other two models. However when enzyme distributions between the four Golgi compartments were also adjusted essentially the same results were achieved with all three models. More recently their model has been extended to handle extracellular metabolites [25].

Liu and Neelamegham [26] developed a computer package, "IBRENA", for simulating and analyzing biochemical reaction networks. IBRENA allows generation of reaction networks connecting specified initial and product glycan pairs. The package was used to model the formation of the sLeX epitope on O-glycans [27]. Best-fit rate constants were determined from experimental glycan structure data and were verified in separate reaction rate measurements.

The KB2005 model was extended (KB2009) to allow unlimited lactosamine repeats and to incorporate more enzymes [28]. A new more general scheme was developed to describe the enzyme reaction rules that specify enzyme action. The improved system can easily incorporate new enzymes and glycan types. A network pruning method was developed to limit the size of the models. A method was added to allow calculation of a complete mass spectrum from the model-predicted glycan distribution. This allows direct comparison of the model result with an experimental mass spectrum with no need for manual annotation. As an example N-glycan mass spectra for normal and malignant human monocytes were analyzed using the model to infer differences in enzyme activities. The model-inferred enzyme activity shifts were in general agreement with literature reported changes in gene regulation between normal and malignant cells. In another study [29] the KB2009 model was used with mass-spectra of human prostate cancer cells of different degrees of advancement. In this case the model-inferred changes in enzyme activity levels were compared with gene microarray measurements for the individual enzyme genes.

More recently Spahn et al. [30] used the same KB2009 enzyme reaction rules for their low-parameter Markov chain modeling approach. Hou et al. [31] also utilized the KB2009 enzyme reaction rule format and glycosylation reaction rules as input to their system for generating glycan structures and reaction networks.

Manipulations of glycosylation in CHO cells offer a potential for improving the desired therapeutic properties of glycoproteins produced by these cells. In that respect Pamela Stanley’s group has developed a set of CHO cell mutants [7,8,9,14,16] for which much more detailed glycosylation patterns have been reported [7]. In this study we use the latest version of the model to infer enzyme activity profiles and glycan structural details from the published MALDI-TOF mass spectrometry. To assist in this process, we demonstrate and discuss the differences in model predicted enzyme activity profiles and glycan profiles for the wild-type Pro¯5 CHO cells vs. 9 mutants of Pro¯5. This work has applications in glycosylation metabolic engineering for therapeutic production from CHO cells. It is especially useful in predicting how the activity of any single glycosylation enzyme or combination of enzymes affects the abundances of glycoforms coded by other enzymes.

It has been observed that in cells where the genes coding for a particular enzyme or group of enzymes are knocked out, compensating up-regulation of other enzymes can occur. For example when mice were genetically modified to eliminate the Mgat2 gene coding for GnTII, the survivors were found to produce a novel glycan with a branch on the bisecting GlcNAc that apparently compensated to some extent for the loss of complex glycans [32].In another mouse study [33,34] the knockout of the Core 2 O-glycan initiated by C2GnT showed compensating production of elongating O-mannose glycans and lactosamine repeats.

More recently a variety of cell types (including CHO cells)with loss of ManII or GnTII activity were grown in culture [35]. The loss of ManII or GnTII activity caused the expected loss of complex N-glycans, but also engendered up-regulation of polyLacNAc chains on the remaining hybrid glycans. The authors suggest that this regulation is needed to stabilize the cell-surface galectin-glycoprotein lattice required for receptor localization and signaling in the complete organism. Further, the studies showed no changes in total enzyme concentration or nucleotide-sugar donor concentration to account for the increased polyLacNAc. Thus the authors proposed that the regulation most likely operates through redistribution of the enzymes and/or nucleotide-sugar donors among the Golgi compartments, with a plausible physical mechanism for how the UDP-GlcNAc shifts to the location of b3GnT (iGnT in this paper) might occur.

Interestingly, the model-predicted enzyme activity shifts presented below suggest that similar compensating regulation could be occurring in the mutant cell lines studied here.

## Results and discussion

### CHO cells included in this study

A wide variety of CHO cell mutants have been isolated by exposing parent populations of CHO cells to various cytotoxic plant lectins, thus selecting mutants with resistance to a given lectin or combination of lectins [8].The mutations often involve defects in genes required to produce specific glycan structures that interact with the lectins chosen, thus resulting in loss-of function mutants. These have been designated using the prefix "Lec". Interestingly other mutants isolated by this procedure produce glycan structures not present in the parent cell population, and are thus gain-of-function mutants. The gain-of-function mutants have been designated with the prefix "LEC". The genetic basis of these gain-of-function mutants, where normally inactive enzyme genes become activated, is largely unknown [8].

More recently, glycans produced by a set of these mutant cell lines, as well as the parent Pro¯5 CHO cells, have been analyzed using a variety of analytical techniques to characterize their glycan structures qualitatively [7]. A set of MALDI mass spectra for the glycans of these cell populations were also submitted to the CFG web site by the authors. We have downloaded these mass spectra and used a KB2009 model-based approach to analyze the mass spectra quantitatively in terms of glycan structures and enzyme activities.

### Use of the model to analyze experimental MALDI mass spectrometric data in terms of enzyme activities

The original KB2005 model developed for CHO cells was migrated to the KB2009 platform to allow generation of synthetic mass spectra and was also extended to allow more lactosamine repeats, branch fucosylation, and α3-galactose termination of branches. Also, a new method has been introduced to specify the distribution of glycosylation enzymes through the Golgi apparatus. Model details are provided in the Methods and Models section.

**Fig 1:**
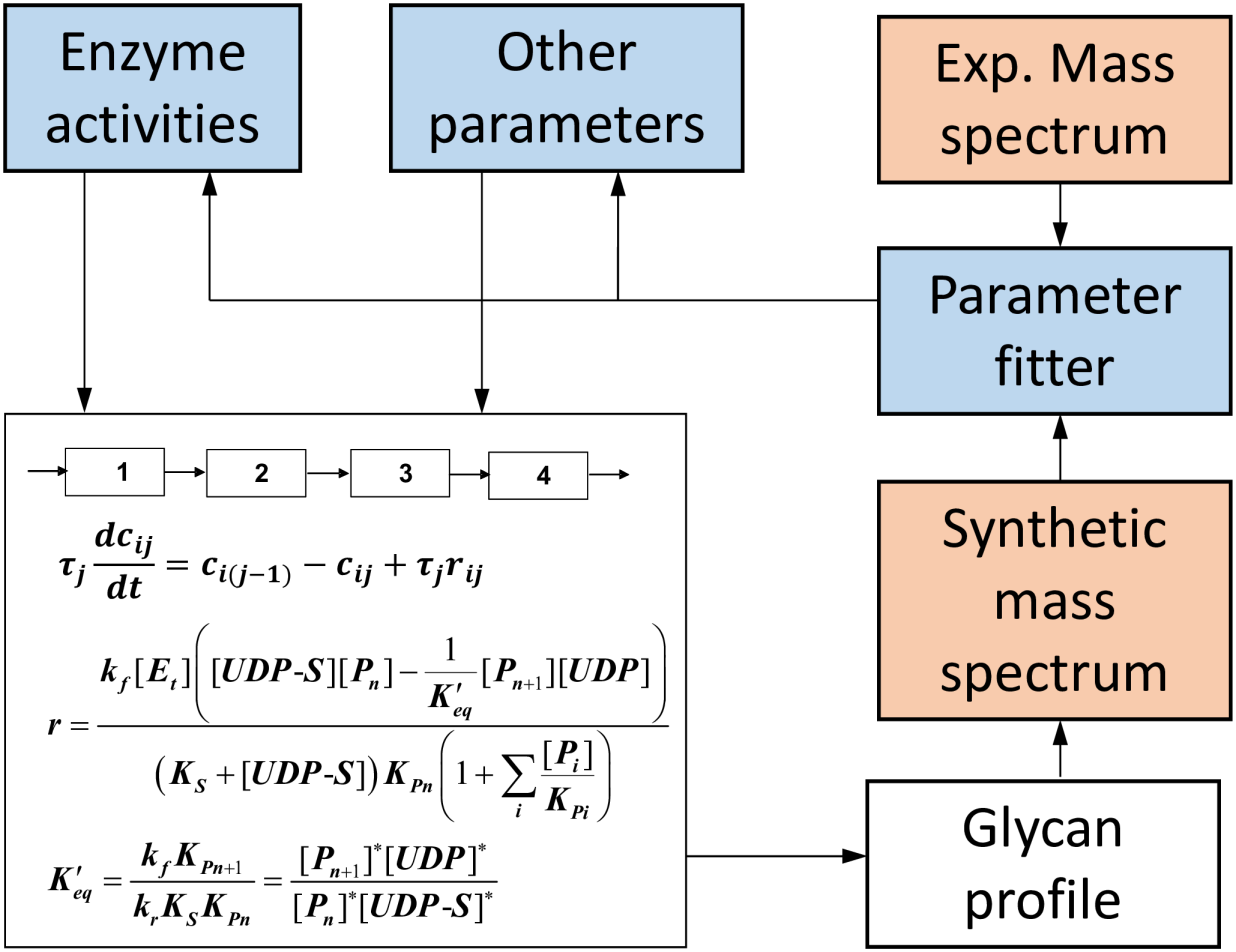
Schematic representation of the CHO N-glycosylation model. At the beginning the N-Glycosylation model applies reaction rules that express enzyme specificity to the initial glycan structures (Man9 and Man8). The result of that is a set of reactions and product glycan structures. Next, the kinetic model –where the Golgi apparatus is modeled as 4 well mixed reactors with a set of enzymes distributed through them –is solved for any set of enzyme concentrations and reaction rate parameters. The model solution results in a complete set of abundances of the glycan structures obtained from the generated glycosylation reaction network. Finally, a synthetic mass spectrum is obtained and compared iteratively to the experimental MALDI-TOF mass spectrum, by a non-linear fitting algorithm that solves the model multiple times by adjusting enzyme concentrations and other parameters each time. To obtain robust estimates of case to case shifts in enzyme activity only total enzyme concentrations are varied between cases, holding all other parameters constant.

A schematic representation and explanation of how the model is used with mass spectrometric data is shown in Fig 1. Solving the model for each case generates estimated abundances for all the glycan structures included in the model (20,000–35,000 structures for the cases studied here). From the estimated abundances, we calculate a synthetic mass spectrum over the range of glycan masses included in the model. Model total enzyme concentrations are then adjusted for each case to bring the calculated mass spectrum into agreement with each measured mass spectrum over the model mass range. Model parameters other than enzyme concentrations may also be adjusted, but only for all cases together, to improve the overall fit to the set of spectra. Only the total enzyme concentrations are changed from one case to another. The end result is an interpretation of the experimental mass spectra in terms of relative enzyme activities, along with detailed estimates of glycan structure abundances for each case.

**Fig 2:**
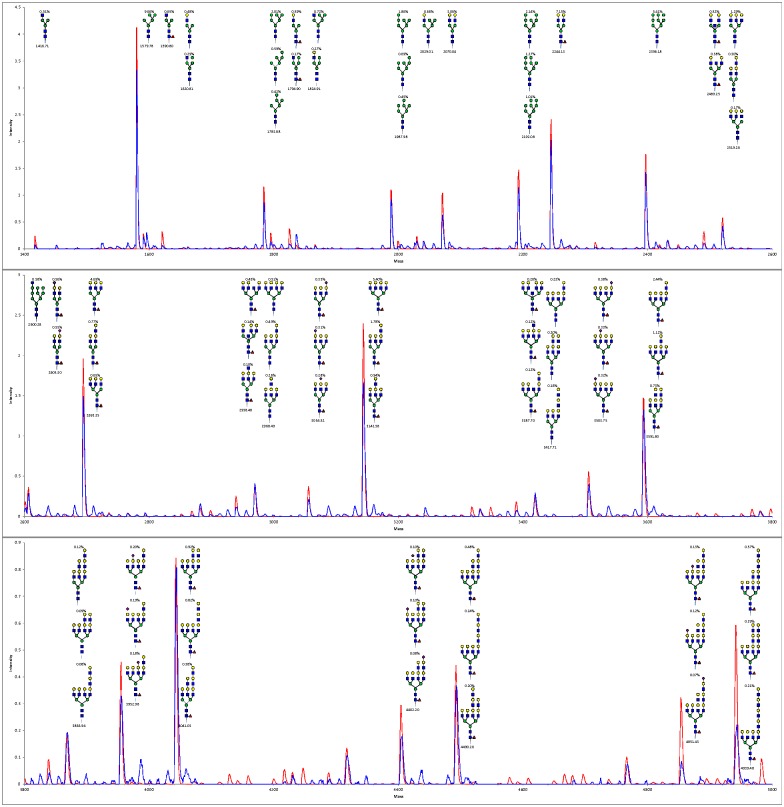
Comparison of measured and model-calculated MS for wild type CHO (Pro¯5). Model enzyme activities are adjusted to bring the model-calculated mass spectrum (red) into agreement with the measured mass spectrum (blue). The "peaks" in this Fig are actually groups of isotopic satellite peaks with their tops connected by straight lines. The calculated spectrum automatically provides identification of the glycan structures within each peak. The three most abundant structures are indicated for each of the larger peaks (≥ 0.5%). The model-calculated abundance of each structure, as percent of the total glycans, is shown above the structure and the monoisotopic mass is shown below each group of structures. There are a number of smaller peaks in the measured mass spectrum that do not correspond to structures included in the model, some of which may be artifacts or serum contaminants. Since the peak intensities of both the measured and calculated spectra are normalized to add up to 100% the measured intensities of individual peaks tend to be lower than the calculated intensities by about 20%.

**Fig 3:**
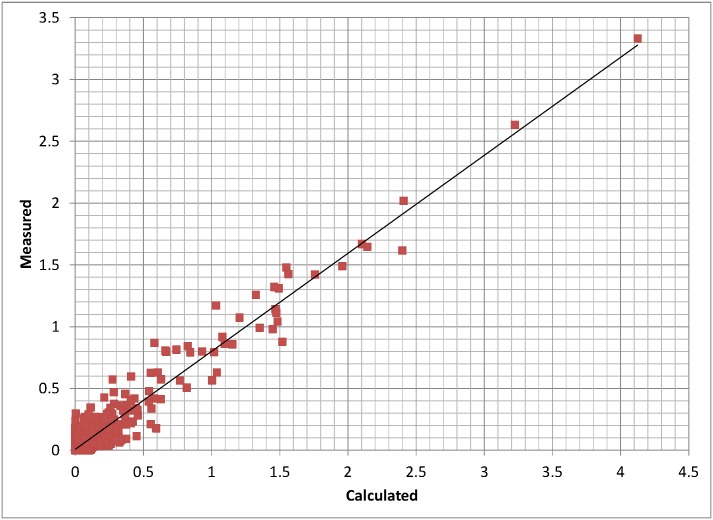
Comparison of measured and calculated individual isotopic mass spectrum peaks for the Pro¯5 CHO cell line shown in Fig 2. The least-squares straight line has a slope less than one because the measured spectrum includes a large number of unknown small peaks (possible artifacts or serum contaminants) that were not included in the model while both the measured and calculated peaks were both normalized to add up to 100%. R-squared = .934.

Figs 2 and 3 compare the model-generated synthetic mass spectrum to the experimental MALDI-TOF mass spectrum for the parent Pro¯5 cell line over the 1400–5000 Dalton mass range included in the model. The overlap between calculated and experimental spectra in Fig 2 and the parity plot of individual peak intensities in Fig 3 indicate good agreement. Similar plots for all the cell lines studied are included as Supporting Information S1 Fig. The model enables identification of distinctive and subtle glycan fingerprint differences between wild type CHO cells and the various mutants. These structures are consistent through the whole range of the spectrum since they result from the enzyme activities adjusted to get good agreement between predicted and experimental spectra.

The adjusted model enzyme activities for all the cell lines studied are shown in Table 1,where the model derived enzyme activity levels of the wild type CHO vs. the nine CHO mutants is compared with qualitative shifts in glycosylation features previously reported [8]. For CHO mutants, which had known losses in expression of various glycosylation enzymes or enzymes involved in sugar-nucleotide transport or synthesis, the reported biochemical shifts were based on those losses of function. In other cases, the reported shifts were based on observed changes in gene activation or glycan profile. Since the current version of the model only includes glycosylation reactions and not sugar-nucleotide transport or synthesis, any change in these latter processes would be reflected in the model glycosylation enzyme activities. In Table 1, yellow highlights denote model enzyme activities expected to change according to previously reported biochemical shifts.

**Table 1:** Model derived enzyme activities for CHO cell lines.

	Pro¯5	Lec1	Lec2	Lec3.2.8.1	Lec4	LEC10	LEC11	LEC12	Lec13	LEC30
Previously reported biochemical change*		↓GnTI	↓CMP-Sia transport	↓Sia synthesis ↓CMP-Sia transport ↓UDP-Gal transport ↓GnTI	↓GnTV	↑GnTIII	↑a3FucT	↑a3FucT	↓GDP-Fuc synthesis	↑a3FucT
ManI	5.032	2.802	5.795	2.156	5.064	5.495	8.643	6.879	5.276	6.980
ManII	8.130	8.659	10.775	4.317	6.793	13.301	8.258	8.390	19.937	21.389
a6FucT	4.161	0.531	5.722	1.807	4.188	10.589	4.331	3.942	0.088	53.310
GnTI	1.049	0.098	1.499	0.173	1.228	1.428	1.325	1.333	1.159	1.907
GnTII	6.582	7.130	8.644	1.456	5.433	13.498	5.803	6.092	20.154	19.659
GnTIII	0.105	0.592	0.201	5.569	0.010	21.569	0.321	0.093	0.081	0.226
GnTIV	0.467	0.145	0.509	2.389	1.181	8.115	0.900	0.317	0.635	0.615
GnTV	2.183	1.530	4.048	8.633	0.409	13.158	3.142	1.784	4.391	4.626
iGnT	0.523	0.000	0.576	0.443	0.439	0.585	0.351	1.214	0.556	0.184
b4GalT	6.282	2.547	8.144	11.553	4.899	5.140	10.174	6.386	7.309	18.330
a3SiaT	0.081	0.179	0.004	0.290	0.047	0.034	0.231	0.068	0.054	0.031
a3FucT	0.006	0.000	0.001	0.248	0.003	0.005	0.078	0.764	0.009	0.567
a3GalT	0.028	0.095	0.007	1.126	0.017	0.011	0.095	0.107	0.032	0.057

Figs in the Table are enzyme activities (k_f_c_enz_/K_m_ min^-1^). The first row also shows the main biochemical changes for each mutant compared to the parent cell population *as reported by Patniak and Stanley[8]. The downward arrows indicate loss of function and the upward arrows indicate gain of function.

Examination of the highlighted entries in Table 1 shows that in most cases the model enzyme activities for the mutant cells move from the parent Pro¯5 activities in the expected directions. The exceptions are two of the three highlighted values in the Lec3.2.8.1 column. These will be discussed below under the Lec3.2.8.1 heading. It should be noted that these results are derived from raw mass-spec data alone without any prior annotation or knowledge of other analytical results. In addition to the expected shifts in enzyme activity the model infers a number of previously unexpected shifts in the glycan enzyme activities of these cell lines. These additional shifts will also be discussed below.

In addition to estimates of enzyme activities, matching the model-calculated mass spectrum to the measured mass spectrum automatically provides a complete annotation of all the mass peaks in the spectrum and a set of all the model glycan abundances. This allows a quantitative estimate of any structural feature of the glycans. A variety of glycan features derived for each of the 10 cell lines is shown in Table 2.

**Table 2:** Abundance of glycan structural features (% of total N-glycans).

Description	Pro¯5	Lec1	Lec2	Lec3.2.8.1	Lec4	LEC10	LEC11	LEC12	Lec13	LEC30
High mannose	26.29	89.16	23.95	85.99	24.87	20.15	22.18	22.33	25.49	13.02
Hybrid	5.38	1.02	3.49	6.19	5.69	9.28	5.23	4.32	2.00	2.65
Monoantennary	2.40	0.46	1.60	2.90	2.53	3.76	2.50	2.13	0.80	1.09
Biantennary	16.44	4.18	10.32	2.30	27.78	25.68	14.72	19.34	9.00	16.18
Triantennary1	1.61	0.11	0.67	0.58	21.63	8.52	2.04	1.70	0.61	0.87
Triantennary2	21.13	4.27	24.25	2.06	3.91	7.62	17.71	25.07	21.02	27.47
Tetraantennary	29.14	1.26	37.31	2.89	16.11	28.76	38.12	27.24	41.87	39.81
Bisected	2.86	2.21	4.93	4.67	0.35	71.13	6.49	2.94	2.15	3.89
Lactosamine extensions	67.10	0.00	90.51	5.10	34.25	37.09	54.86	67.99	82.87	34.45
Lactosamine groups	277.86	25.04	331.36	26.45	225.72	230.36	255.98	168.56	323.08	92.63
Terminal α-galactose	3.27	1.55	0.91	5.83	1.93	1.07	9.90	4.33	4.21	4.21
Terminal sialic acid	16.71	9.55	0.94	8.59	9.60	6.86	46.24	10.71	11.71	5.37
Core Fucose	58.22	3.04	65.86	8.23	61.81	63.83	59.53	60.78	1.63	86.36
Branch fucose	3.42	0.02	0.60	7.45	1.62	1.92	40.56	122.09	5.70	219.77
Lex	3.06	0.01	0.59	4.35	1.52	1.87	28.20	63.72	5.25	79.03
SLex	0.20	0.01	0.00	1.29	0.06	0.03	5.12	2.14	0.19	3.06
VIM-2	0.00	0.00	0.00	0.01	0.00	0.00	0.03	0.18	0.00	0.18

Structures were derived by fitting the model to match the raw glycan mass spectra from each of the CHO cell lines. The various substructures in the table are defined by the following codes: High mannose, #GN = 2; Hybrid, #GN>2 & ~GNb2|Ma6; Monoantennary, GNb2Ma3|(Ma6)Mb4; Biantennary, GNb2Ma3|(…GNb2Ma6)Mb4; Triantennary1, GNb2(…GNb4)Ma3|(…GNb2Ma6)Mb4; Triantennary2, GNb2Ma3|(…GNb2(…GNb6)Ma6)Mb4; Tetraantennary, GNb2(…GNb4)Ma3|(…GNb2(…GNb6)Ma6)Mb4; Bisected, Ma3(GNb4)(…Ma6)Mb4; Lactosamine extensions, Count GNb3; Lactosamine groups, Count Ab4GN; α-galactose, Count (Aa; Sialic acid, Count (NN; Core Fucose, GNb4(Fa6)GN; Branch fucose, Count Fa3; Lex, Count (Fa3(Ab4)GN; SLex, Count (Fa3(NNa3Ab4)GN; VIM-2, Count (Fa3(NNa3Ab4GNb3Ab4)GN.

#### Lectin sensitivity and resistance of the cell lines

The detailed procedures used to produce the mutant strains of CHO were quite variable, involving mutagenic agents and one or more lectins, applied at once or in sequence, to produce cells with varied combinations of resistance or sensitivity to the various lectins. Table 3 lists the cell lines used in this study and their relative sensitivities to a panel of lectins, including the lectins used to select the cell lines. The relative sensitivities of each cell line compared to the parent Pro¯5 cells are based on the concentration of each lectin required to reduce plating efficiency of each cell line to 10%, as reported by Patnaik & Stanley [8]. These sensitivities should correlate with the abundance of the glycan substructures bound by the lectins.

**Table 3:** Relative sensitivity of mutant CHO cells to a panel of cytotoxic lectins.

	Cell lines									
Lectins	Pro¯5	Lec1	Lec2	Lec3.2.8.1	Lec4	LEC10	LEC11	LEC12	Lec13	LEC30
L-PHA	1	0.001	1.5	?	0.001	2	0.25	0.33	1	0.10
WGA	1	0.03	0.09	?	0.67	1.5	0.13	0.02	1	0.02
ConA	1	6	1	?	1.5	1	1	1	1	0.67
Ricin	1	0.01	100	?	1.5	0.05	25	4	1	1.5
LCA	1	0.01	2	?	1.5	1	0.33	0.50	0.04	0.25
PSA	1	0.11	2	?	2	1	1	1	0.02	?
E-PHA	1	0.10	1	?	0.67	10	0.67	?	?	?
MOD	1	0.25	5	?	1	1	0.50	0.25	1	?
Abrin	1	0.003	10	?	?	0.05	5	1.5	1.5	?

The table shows relative sensitivities of the cell lines based on the lectin concentration required to reduce plating efficiency to 10%, as reported by Patnaik and Stanley [8]. L-PHA, leukophytohemagglutinin from Phaseolus vulgaris; WGA, wheat germ agglutinin; ConA, concanavalin A; Ricin, Ricinus communis lectin II; LCA, Lens culinaris lectin; PSA, Pisum sativum lectin; E-PHA, erythrophytohemagglutinin from Phaseolus vulgaris; MOD, modeccin, Adenia digitata; Abrin, Abrus precatorius.

The sensitivities of the cell lines to the lectins depends on the presence of specific glycan substructures bound by the lectins within the cell glycans. Bound glycan substructures for a number of the lectins have been summarized by Cummings and Etzler [36] and more details can be found in Cummings [37] and Debray et al. [38]. Any given lectin may bind a variety of substructures with more or less affinity and the affinity can be modified by the presence or absence of other substructures on the complete glycan. For each glycan the highest affinity substructures were chosen to analyze the model-predicted glycans for each cell line. Using the model-predicted detailed N-glycan structures for each cell line the calculated abundances of the lectin-bound substructures are given in Table 4.

**Table 4:** Model-predicted abundances of glycan substructures bound by various lectins within each cell line.

	Cell lines									
Lectins	Pro¯5	Lec1	Lec2	Lec3.2.8.1	Lec4	LEC10	LEC11	LEC12	Lec13	LEC30
L-PHA	37.5	1.6	57.1	0.7	16.0	24.9	22.8	10.1	49.6	6.6
WGA	16.5	9.5	0.9	7.3	9.5	6.8	41.0	8.6	11.5	2.3
ConA	28.3	89.5	25.2	87.4	26.9	21.0	23.9	23.8	26.3	14.1
Ricin	191.1	13.9	239.0	9.2	180.0	185.4	151.6	92.2	224.6	73.7
Ricin(F)	194.2	14.0	239.6	14.4	181.6	187.3	185.5	207.6	230.0	268.4
LCA	27.2	2.3	28.0	1.6	24.4	25.2	15.2	6.8	0.4	4.3
PSA	27.2	2.3	28.0	1.6	24.4	25.2	15.2	6.8	0.4	4.3
E‐PHA	1.1	0.9	1.3	1.3	0.2	24.6	2.2	0.4	0.5	0.4

Substructure codes selected for the analysis: L-PHA, (Ab4…GNb2(Ab4…GNb6)Ma6; WGA, Count (NNa3Ab4GN; ConA, Ma3(…Ma3(…Ma6)Ma6)Mb4; Ricin, Count (Ab4GNb; Ricin(F), Count (Ab4|GNb; LCA & PSA, -GNb2Ma3|(-GNb2(-GNb6)Ma6)Mb4GNb4(Fa6)GN or -GNb2Ma3|(-GNb2Ma6)Mb4GNb4(Fa6)GN; E-PHA, GNb2|Ma3(GNb4)(-GNb2Ma6)M. The use of codes to specify substructures is explained in the Methods and Models section.

We may compare the lectin-bound substructure abundances in Table 4 with the relative sensitivities of the cell lines to each lectin in Table 3. For the most part, aside from the ricin results, the two tables are directionally similar. For ricin, the binding substructure is a terminal lactosamine group. It isn't clear from the references [36,37,38] how the binding of ricin to the lactosamine groups is affected by addition of fucose to the GlcNAc of the lactosamine group. Table 4 includes two entries for ricin labeled "Ricin" and "Ricin(F)" which exclude or include, respectively, lactosamine groups with fucose added. The very small abundance of lactosamine groups in Lec1 cells explains the low sensitivity of Lec1 cells to ricin, but terminal lactosamine groups are quite prevalent in the LEC10 cells, which also have very low sensitivity to ricin. Apparently the high abundance of bisecting GlcNAc groups in LEC10 cells (see the "Bisected" row of Table 2) interferes with the binding of ricin to the lactosamine groups. Also the sensitivities of LEC11, LEC12 and LEC30 cells may correlate with some weighted average of the Ricin and Ricin(F) rows in Table 4.

The results for lectins LCA and PSA in Table 4 are identical because the lectins bind to the same substructure on the glycans. However Table 3 shows that they have different specificities for some of the cell lines. Other discrepancies between Tables 3 and 4 are discussed below under "Model-based analysis of each cell type".

### Other model parameters and assumptions

In addition to adjusting the total enzyme concentrations for each cell line to match each of the individual experimental mass spectra, other model parameters were adjusted for all the cell lines at once to improve the overall matching of the mass spectra. Such adjustments were only made when they provided a clear improvement in the accuracy of the predicted mass spectra, so many parameters were left unchanged from previous versions of the model.

#### Spatial distribution of enzymes

In the originalKB2005 version of the model [4] the spatial distribution of enzymes was based on the distribution assumed in theUB1997 Umana and Bailey model [19], except for the distribution of the iGnT enzyme, which had not been included in the UB1997 model. The assumed distributions for ManI, ManII and GnTI—GnTV were (0.15, 0.45, 0.3, 0.1) across the four compartments and (0.0, 0.05, 0.2, 0.75) for b4GalT and SiaT. Since iGnT can only act on galactosylated glycans it was moved out of the first compartment, which has no b4GalT, into the second, to give (0.0, 0.6, 0.3, 0.1). This set of distributions worked well for the data analyzed with the KB2005 model, which had negligible high-mannose glycans. In the data analyzed with the KB2009 model for normal human monocytes and monocytic leukemia cells [28] and for prostate cancer cells [29], there were significant amounts of high-mannose glycans. In these case a good fit to the data required moving much of the ManI enzyme into the first compartment.

**Fig 4:**
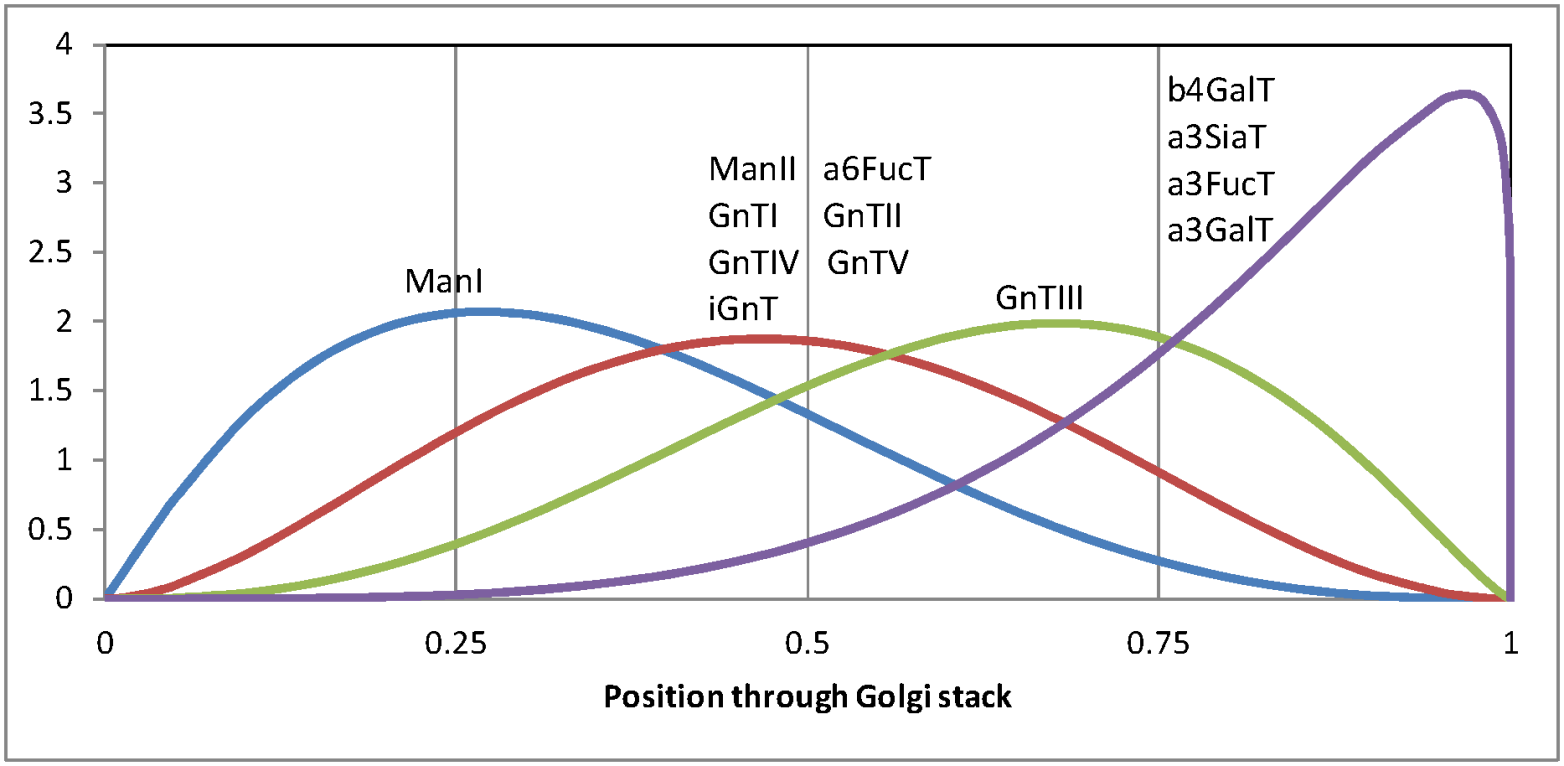
Beta distributions used for spatial distribution of enzymes. The enzymes are distributed among the compartments according to beta distributions with a common value of α + β = 6. The enzymes following each distribution are shown on the curves.

The set of 10 cell lines analyzed in the current paper includes substantial variations in the activities of many of the enzymes and may help to define how the enzymes may be distributed. Thus the latest model version used in the current study includes a method to allow continuous adjustment of the enzyme distributions to best match the glycan mass spectral data. It was found that the original two spatial enzyme distributions used in the UB1997 model could be closely matched using beta distributions with the fraction of enzyme in each compartment corresponding to the probabilities for the intervals (0, 1/4, 1/2, 3/4, 1). The shape parameters of the distribution, α and β, were set by varying the mean of the distributions, μ = α/(α + β), while holding the sum of the parameters constant (α + β = 6).The mean values of these distributions over the complete Golgi stack provides a single continuous variable for each enzyme to adjust for a best match to the experimental data. It was found assuming two of the enzymes, ManI and GnTIII, to distribute separately from the other two groups of enzymes improved the overall matching of the mass spectra. The mean values μ that best matched all of the mass spectra, on a zero to one scale were: for ManI μ = 0.346; for ManII, GnTI, GnTII, GnTIV, GnTV and iGnT μ = 0.479; for GnTIII μ = 0.618; and for b4GalT,a3SiaT, a3FucT and a3GalTμ = 0.812. Varying α + β did not improve the matching of the data. The resulting distributions for the four model compartments are shown in Table 5 and the underlying beta distributions are shown in Fig 4.

**Table 5:** Spatial distribution of enzymes.

	Beta parameters		Compartments			
Enzymes	Mean	α + β	1	2	3	4
ManI	0.346	6	0.340	0.453	0.188	0.019
ManII a6FucT GnTI GnTII GnTIV GnTV iGnT	0.479	6	0.126	0.419	0.371	0.085
GnTIII	0.618	6	0.029	0.237	0.465	0.269
b4GalT a3SiaT a3FucT a3GalT	0.812	6	0.002	0.041	0.244	0.713

The enzymes are distributed among the compartments according to beta distributions with a common value of α + β = 6. The mean values of the distributions were adjusted to give the best fit to the mass spectra for all the cell lines together. Beta distributions are shown in Fig 4.

#### Kinetic parameters and adjustments

The only kinetic parameters that were changed from previous versions of the model are the adjustment factors for the ManI enzyme. This was necessary because of the very high abundances of high-mannose glycans for some of the cell types. These are given in the Methods and Models section.

### Sensitivity of results to model assumptions and parameters

The current study produces two types of results from the mass spectral data for each cell line: an estimate of the abundance of each glycan structure in the model and a predicted set of activities of the model enzymes that explain the structures. When the model can produce a synthetic mass spectrum that accurately matches the experimental one, the estimated glycan abundances should be fairly accurate. The model just provides an automated method of annotating the peaks in the mass spectrum. Compared to a manual annotation of the mass-spectrum peaks the model-based annotation provides additional breakdowns of isomers within ambiguous peaks. It has been found that the model predictions of glycan structural features are fairly insensitive to model assumptions, provided that the model can accurately simulate the measured mass spectrum.

The enzyme activity predictions are sensitive to various model assumptions, such as compartment residence times and the spatial distribution of enzymes through the compartments. Thus the analysis focuses on shifts in enzyme activities between different cell types based on the assumption that everything in the model except the total concentration of each enzyme is unchanged from one case to another. This greatly reduces the sensitivity of the results to various assumed model parameters.

#### Uniform processing of glycans

There are variations in glycan profile between different glycoproteins from the same cell line and even between different glycosylation sites on the same protein. For example, recombinant t-PA secreted from CHO cells has three glycosylation sites, one of which contains only high-mannose glycans while the other two contain only complex glycans [39]. Apparently one glycosylation site on t-PA provides restricted accessibility to the GnTI enzyme. The complex glycans on the other two sites also exhibit some differences in glycan structure between the sites. It has also been found that glycoproteins destined to reside in lysosomes pick up a mannose-6-phosphate marker in the ER that protects high-mannose glycans further processing in the Golgi complex [40]. In this study there is an additional source of glycan variability due to the use of whole cell extracts to produce the glycan samples. These whole cell extracts include incompletely processed glycoproteins from the ER and early Golgi compartments. Such glycoproteins contain additional high mannose glycans compared to fully processed glycoproteins contained in secreted glycoproteins or non-ER or non-Golgi glycoproteins in the cell.

In this model only one type of glycosylation site has been assumed so that all the modeled glycans are exposed to the same sequence of glycosylation enzyme activities in the Golgi apparatus. The objective of the model is not to describe in detail the glycan profile of every individual glycosylation site on every individual glycoprotein in a sample but to model the overall result for a relevant population of sites in a given context. Depending on the context, the relevant population of sites could be a single site on a single glycoprotein, multiple sites on a single glycoprotein, all the sites on all plasma membrane glycoproteins, or, as in this study, all the sites on all the glycoproteins in a whole cell extract. The objective is to understand how changes in enzyme activities can account for the changes in overall glycan profile of whatever population of glycosylation sites is under study. In effect the model simulates a diverse population of glycosylation sites with an average site that matches the overall glycan profile of the population of sites. For example the presence of some sites in the population with only high-mannose glycans can be accommodated by a single site with a somewhat lower average activity of GnTI. We are only predicting how changes in overall enzyme activities affect the overall glycosylation pattern, not the absolute level of the activities.

One could envision that the large number of glycan mass spectrum peaks caused by the great variability observed in glycan structures might require multisite models to capture that detail, but the results above demonstrate that the glycan mass spectrum for each individual cell line can be matched with only a single glycosylation site model, and that the changes in measured spectra between the various CHO cell lines can be matched by changing only the enzyme activities in the model. Furthermore it has been seen that the model-predicted enzyme activity changes are consistent with previously published findings on these cell lines. Thus there is no indication that more detailed modeling is required to make the connection between overall enzyme activities and overall glycan profiles for the whole cell extracts considered here.

#### Four well-mixed reactors in series

Following the original Umana and Bailey model [19] the physical transport within the Golgi network has been approximated by four well-mixed reactors in series. This was based on the picture of the Golgi compartments as static containers with substrate proteins carried forward from one compartment to another by budding vesicles while the glycosylation enzymes and other Golgi-resident proteins are returned to their compartment via retrograde vesicle traffic, thus remaining fixed in their compartments. The four compartments represent the cis-, medial- and trans-Golgi cisternae and the trans-Golgi network. More recent observations have suggested that in fact the Golgi compartments may be dynamic (cisternal maturation model), with new compartments forming at the cis end of the network and moving toward the trans end, carrying the glycoprotein substrates with them. The enzymes are then carried backwards from one compartment to the previous one by selective vesicles. Based on this picture it has been suggested [23,24] that a plug flow reactor model with the enzymes distributed along the reactor length might be a more accurate representation of the Golgi apparatus. Of course either the well-mixed reactors in series or the plug flow reactor is an idealization. If the back-transporting vesicles are not perfectly selective for Golgi resident proteins (enzymes and transporters) they may also cause some backmixing of the substrate glycoproteins. In this case some number of well-mixed reactors in series may actually be a better approximation for the cisternal maturation concept than ideal plug flow.

In any event, it is likely that either model can be brought into agreement with data by adjusting model parameters within a reasonable range. A larger number of well-mixed compartments could be considered, however, which would move the reactors-in-series model closer to the plug flow model. As mentioned in the introduction, Jimenez del Val et al. [24] have shown that by adjusting a larger number of model parameters the four tanks in series model can be made to match the plug flow model results.

As long as the characteristics of the well-mixed reactors in series or the plug flow reactor are unchanged between cases each model will likely give similar results for relative enzyme activities between the cases if they can be brought into agreement with the detailed mass spectral data.

#### Distribution of enzymes and transporter proteins among the compartments

A more significant issue is how the Golgi-resident proteins, enzymes and transporter proteins, are distributed among the compartments. As described above, these spatial distributions have been adjusted to best match the experimental mass spectra for all the cell lines. Adjustments to match the shifts from one cell line to another have been to the total enzyme concentrations, keeping the distribution of each enzyme among the compartments fixed for all the cell lines. This makes model-estimated enzyme activities relatively insensitive to the assumed distributions in most cases, but see the LEC10 discussion below.

#### Kinetic parameters

Mass spectral data has a high dimensionality, typically 300 or more separate monoisotopic masses in each spectrum, and can therefore be effective for determining multiple model parameters simultaneously. However the model has quite a few parameters, many of which were originally derived from literature in-vitro studies on entirely different cell lines, species, substrates, etc.. How sensitive are the model results in this paper to the values of these parameters?

The model parameters that were initially derived from literature sources include the base values of kinetic parameters for each enzyme, adjustment multipliers, total enzyme concentrations, enzyme distributions across the compartments, compartment residence times, and total glycan concentration. Obviously they all affect an individual model calculation of a glycan profile. However the way the model is actually used in this paper is to adjust the total enzyme concentrations for each case to match each mass spectrum while changing a few other parameters for all the cases, usually adjustment multipliers, to improve the overall fit to the data. The reason that most of the model parameters remain unchanged from previously assumed values is that changing them doesn't improve the overall fit. For example the base values of the rate coefficients, k_f_, have no effect because they are multiplied by the total enzyme concentrations which are adjusted to fit each case. The K_md_ values in this study don't matter because the donor cosubstrate concentrations have been fixed. As a test the base values of K_m_ for all the enzymes were arbitrarily changed to a single value of 500 μM. The model was then refit to each mass spectrum by adjusting only the total enzyme concentrations. At the end, the fits of the model to the mass spectra were slightly degraded but the model total enzyme activities for each case (= k_f_c_enz_/K_m_) changed only slightly from Table 1 and the estimated glycan feature abundances hardly changed at all from Table 2 (data not shown). Thus in this case the methodology eliminated the sensitivity of the results to these particular parameters.

#### Aggregation of isozymes

Many glycosylation enzymes are encoded by more than one gene and the different versions, or isozymes, can have different selectivities and/or reaction kinetic parameters. Rather than treating the different isozymes as separate enzymes we have aggregated each isozyme group into a single enzyme. Treating the isozymes separately would, of course, introduce the concentrations of each of the isozymes as additional model variables as well as the kinetic parameters and adjustment rules for each of the isozymes. Since the relative concentrations of the isozymes are generally unknown anyway, we believe there is little loss in accuracy by utilizing adjustment rules for the whole isozyme group to handle both the relative isozyme concentrations and any differences in selectivity of particular isozymes.

### Model-based analysis of each cell type

#### Pro¯5 (wild-type)

Previous observations on wild-type CHO cells [8] have shown a range of complex and high-mannose structures with few hybrid structures. The complex glycans include biantennary, triantennary and tetraantennary structures. The wild-type CHO have also been found to express little or no branch fucosyltransferases (a3FucT) or GlcNAc transferase III (GnTIII), which transfers the bisecting GlcNAc to the glycans. More recently North et al. [7] using a variety of data types, including higher mass MS (up to 11,000 Daltons) MS/MS and GC/MS have shown quite long chains of polylactosamine repeats and the presence of some bisecting GlcNAc structures. The model-based activity estimates for the Pro¯5 wild type CHO cells in Table 1 are consistent with these observations. This is also reflected in Table 2, which predicts small amounts of branch fucose and Le^x^ epitopes. The model also predicts the presence of some hybrid glycans.

#### Lec1

Lec1 cells exhibit a mutation of the Mgat1 OR that results in a loss of GnTI activity, which is needed to synthesize complex or hybrid N-glycans [41].The model-predicted enzyme activities for Lec1 in Table 1 show very low GnTI activity, equal to 0.10, compared to the parent Pro¯5 value of 1.05. Additionally, the calculated abundances of glycoforms shows approximately 90% of the N-glycans to be of the high mannose type (Table 2).Of course if GnTI activity were completely absent in the cells the glycans would be 100% high-mannose. However, the mass spectrum for the Lec1 mutant cells clearly shows the presence of peaks corresponding to complex N-glycans. North et al. [8] suggest that this may be due to traces of serum glycoproteins from the growth medium. There is also the possibility that the cell culture still retains low levels of the parent cell genes. Regardless of the reason for the presence of the complex N-glycans, the model interprets this as indicating some GnTI activity, along with a variety of other glycosylation enzyme activities.

Comparing the entries for Lec1 in Tables 3 and 4 shows that the model-predicted abundances of lectin-sensitive substructures are consistent with the measured lectin sensitivities for Lec1 cells.

#### Lec2

Lec2 CHO cell mutants have a mutation in the Slc35a1 ORF and are unable to transport CMP-sialic acid into the Golgi apparatus, resulting in markedly reduced levels of sialylation [42].

Fitting the model to the mass spectrum of theLec2 cell line generates an enzymatic activity profile which is similar to the CHO Pro¯5 model (Table 1), but with a very low activity of α2,3 sialyltransferase (a3SiaT) and some increase in GnTV activity. While the model does not currently include the nucleotide-sugar transporters, the very low a3SiaT activity has essentially the same effect on the glycan structures as very low transporter activity. This is reflected in Table 2, predicting only 0.1% of sialylated glycans. The model-predicted abundance of lactosamine groups is higher than Pro¯5 due to the lack of sialic acid capping of the glycan branches, thus allowing more lactosamine extensions. The higher predicted GnTV activity also contributes to this by providing more tetraantennary structures to be sialylated. The predicted increase in GnTV activity could be an example of up-regulation of lactosamine to compensate for the loss of sialic acid on the glycan, similar to studies described in the introduction [32,35].

Comparing the entries for Lec2 in Tables 3 and 4 shows that the model-predicted abundances of lectin-sensitive substructures are consistent with the measured lectin sensitivities for Lec2 cells, except that the measured sensitivity to ricin shown in Table 3 seems disproportionately high compared to the increase in abundance of the ricin binding site from the parent Pro¯5 cells shown in Table 4.

#### Lec3.2.8.1

Lec3.2.8.1 carries four glycosylation mutations corresponding to insertions/deletions in the *Gne*, *Slc35a1*, *Slc35a2*, and *Mgat1*ORFs. Impairment of these genes results in loss of sialic acid synthesis activity, CMP-Sia transport activity, UDP-galactose transport activity, and GnTI activity, respectively [43]. As shown in Table 1, the model-based enzyme activities predict the expected drop in GnTI activity, resulting in the large amount (86%) of high-mannose glycans shown in Table 2, but no drop in a3SiaT activity, as expected from a reduced level of CMP-Sia transporter, or b4GalT activity, from a reduced level of UDP-Gal transporter. In fact, they both moved in the opposite direction to higher levels than the parent Pro¯5 cells. Compared to the model-calculated enzyme activities for the parent Pro¯5 cells, the Lec3.2.8.1 cells show lower GnTII activity, allowing the Lec3.2.8.1 cells to maintain a similar abundance to the parent cells of hybrid N-glycans in spite of the reduced GnTI activity. We also see higher GnTIII, GnTIV, GnTV and a3GalTactivities compared to the parent cells. Again North et al.[8] note that this cell line should only contain high-mannose N-glycans and attribute the presence of the complex glycans indicated in the mass spectrum for the Lec3.2.8.1 cells, as well as the Lec1 cells, to possible serum glycoprotein contamination. This would explain the model predictions of enzyme activities needed to produce the small amounts of complex glycans.

#### Lec4

Lec4 cells have a deletion in the Mgat5ORF causing loss of GnTV activity, which is needed to initiate the GlcNAc β(1,6) Man α(1,6) branch of the glycan[44].As expected, the model derived enzyme activities for Lec4 predict a large drop in GnTV activity, by about a factor of 5. However, the model also predicts a significant increase in GnTIV activity, by about a factor of 3, which was not expected. These model-derived shifts lead to the structural changes shown in Table 2. Compared to the parent Pro¯5 cells, we see a large increase in triantennary 1 structures (due to higher GnTIV), a large decrease in triantennary 2 structures (due to lower GnTV), a 50% increase in biantennary structures, and a 40% drop in tetraantennary structures. The predicted increase in GnTIV activity could have resulted from up-regulation of GnTIV in the mutant cells to compensate for the loss in GnTV activity and reduce the loss of lactosamine groups. Given that triantennary 1 and triantennary 2 structures occur in pairs with identical masses it is remarkable that the model can predict this shift from the mass spectrum data alone. This finding could illustrate an ability to extract information from mass spectral data not normally available from mass spectra alone.

Comparing the entries for Lec4 in Tables 3 and 4 shows that the model-predicted abundances of lectin-sensitive substructures are consistent with the measured relative lectin sensitivities for Lec4 cells, but the drop in abundance of the L-PHA sensitive substructure for Lec4 in Table 4 does not seem large enough to explain the almost complete lack of sensitivity of Lec4 to L-PHA shown in Table 3. This suggests that the drop in GnTV activity is larger than the model predicts.

#### LEC10

LEC10 cells exhibit activation of theMgat3 gene which provides GnTIII activity [45]. This activity adds a bisecting GlcNAc to the glycan. As expected, the model-predicted enzyme activities for the LEC10 mutant cells in Table 1 clearly include a very large increase in GnTIII activity. In addition, the model shows significant increases in the activities of ManII, a6FucT, GnTII, GnTIV and GnTV. These latter shifts were not expected. The shifts occur because once a glycan becomes bisected by the GnTIII enzyme, the ManII, a6FucT, GnTII, GnTIV and GnTV enzymes can no longer act on that glycan. In fact the LEC10 results in Tables 2 and 4 show only minor changes in the complex glycans produced by these enzymes. The large increases in the activities of these enzymes in the model allow them to act before the glycans become bisected.

Aside from a compensating regulatory increase in all these enzymes together, the lack of effect of increased GnTIII activity on the complex glycan abundances could also be explained by a distribution of the GnTIII enzyme across the Golgi compartments different from that assumed in the model. If the GnTIII enzyme occurs later in the sequence of model compartments, the enzymes suppressed by bisecting GlcNAc have more time to act before the glycans become bisected.

The distribution of GnTIII originally used in the model dates back to the original UB1997 model [19]. The assumed enzyme distributions were based on a number of literature studies. Lacking any literature data on how GnTIII is distributed across the Golgi compartments, the authors assumed that it is distributed identically to ManII, GnTI, GnTII, and GnTIV which had been measured at that time in a variety of cell types. The same assumption was made for ManI and GnTV. Since that time only the ManI enzyme distribution used in the model was changed in KB2009 to better match data on high-mannose glycans. As discussed above, in the current version of the model both ManI and GnTIII have been adjusted to new distributions common to all the cell lines to give better fits to the mass spectra.

To test the possible effect of GnTIII distribution on the results for LEC10, the model was fit to the Pro¯5 and LEC10 mass spectra with a spatial GnTIII distribution equal to that of b4Galt (see Table 5 and Fig 4).Readjusting the total enzyme concentrations to match the measured mass spectra for both Pro¯5 and LEC10 resulted in changes in the model-predicted enzyme activities and glycan feature abundances shown in Table 6.

**Table 6:** Effect of GnTIII spatial distribution on model-predicted enzyme activities and glycan features for parent Pro¯5 cells and LEC10 cells.

GnTIII distribution	Best fit		GnTIII = b4GalT		GnTIII distribution	Best fit		GnTIII = b4GalT	
Enzyme activities	Pro¯5	Lec10	Pro¯5	Lec10	Glycan features	Pro¯5	Lec10	Pro¯5	Lec10
ManI	5.032	5.495	5.030	5.500	High mannose	26.29	20.15	26.30	20.19
ManII	8.130	13.301	8.002	14.299	Hybrid	5.38	9.28	5.43	9.19
a6FucT	4.161	10.589	4.080	10.298	Monoantennary	2.40	3.76	2.44	3.58
GnTI	1.049	1.428	1.049	1.429	Biantennary	16.44	25.68	16.48	25.69
GnTII	6.582	13.498	6.406	14.896	Triantennary1	1.61	8.52	1.60	9.22
GnTIII	0.105	21.569	0.227	57.734	Triantennary2	21.13	7.62	21.14	4.74
GnTIV	0.467	8.115	0.440	6.601	Tetraantennary	29.14	28.76	29.04	30.98
GnTV	2.183	13.158	2.092	6.586	Bisected	2.86	71.13	2.38	71.59
iGnT	0.523	0.585	0.538	0.586	Lactosamine extensions	67.10	37.09	67.52	39.32
b4GalT	6.282	5.140	6.044	5.096	Lactosamine groups	277.86	230.36	277.47	233.91
a3SiaT	0.081	0.034	0.080	0.035	α-galactose	3.27	1.07	3.27	1.15
a3FucT	0.006	0.005	0.006	0.006	Sialic acid	16.71	6.86	16.66	7.11
a3GalT	0.028	0.011	0.028	0.012	Core Fucose	58.22	63.83	58.19	64.71
					Branch fucose	3.42	1.92	3.45	2.22

The results with the best-fit distribution of GnTIII (used in Tables 1 and 2) are compared with the results using a distribution of GnTIII the same as the distribution of b4GalT (Table 3 and Fig 4). This moves the GnTIII enzyme to later compartments in the model. The magnitude of the model-predicted enzyme activity changes for the LEC10 cells relative to the parent Pro¯5 cells are somewhat different for the different GnTIII distribution but the model-predicted glycan feature changes are only slightly affected. (See Table 2a for definitions of glycan features.)

As the GnTIII enzyme is moved to later compartments the overlap between GnTIII and the enzymes it suppresses is reduced. However GnTIII activity is itself suppressed by the presence of galactose on its substrate, so GnTIII needs to begin acting before galactosylation has progressed too far. Table 6 shows that while the change in distribution of GnTIII had a negligible effect on the model-predicted enzyme activities or glycan feature abundances for the base Pro¯5 cells, the change had some effect on the model-predicted enzyme activities for the LEC10 cells. With the GnTIII distribution set equal to the distribution of b4GalT, the predicted increases in the activities of the enzymes suppressed by the presence of bisected glycans (ManII, a6FucT, GnTII, GnTIV and GnTV) between Pro¯5 and LEC10 were less than the increases predicted with the best fit distribution. At the same time the predicted increase in GnTIII activity was greater with the b4GalT distribution because of the presence of galactosylated substrates in the latter compartments. The predicted abundances of glycan features for the LEC10 results changed only slightly with the change in GnTIII distribution, mainly in the split between triantennary and tetraantennary glycans. It was also found that shifting the distribution of GnTIII still later in the compartments made the match to the mass spectral data poorer (not shown). Thus even with a modified distribution of GnTIII, still consistent with matching the LEC10 mass spectrum, the model predicts increases in the enzymes suppressed by bisection of the glycans to maintain the number of lactosamine groups in the glycans. Again, this could be due to genetic changes in the LEC10 cells or regulatory effects.

Except for ricin, the model-predicted shifts in abundances of lectin-bound substructures for LEC10 in Table 4 are in reasonable agreement with the lectin sensitivities in Table 3. As mentioned previously, the large increase in bisected structures apparently eliminates the sensitivity of the LEC10 cells to ricin.

#### LEC11

LEC11 cells are gain-of-function mutants of the parent Pro¯5 cells that express an α-1,3-fucosyltransferase (a3FucT) caused by activation of the Fut6A or Fut6B gene, in turn caused by loss of a regulatory factor [46].Referring to the model results in Table 1, we see that LEC11 is predicted by the model to have the expected presence of a3FucT activity compared to a negligible activity in the parent Pro¯5 cells. We also see a significant increases in GnTIV, GnTV, a3SiaT and a3GalT, changes which were not expected.

The predicted shifts in glycan structural features are shown in Table 2. We see the glycan branch fucosylation increasing from about 3% in the parent Pro¯5 cells to about 40% in the LEC11 cells. Tetraantennary glycans increase due to the higher GnTIV, and sialic acid increases from about 17% to 46%, due to both higher a3SiaT activity and more available sites for sialylation on the tetraantennary glycans. These model-predicted shifts would be consistent with up-regulation to compensate for loss of lactosamine due to fucosylation of the lactosamine groups. Also, SLe^x^ structure increases from a negligible amount to about 5%. The model also predicts the presence of about 0.04% of the VIM-2 structure and an increase in α-galactose to about 10% compared to 3% for the parent cells.

Comparing the entries for LEC11 in Tables 3 and 4 shows reasonable agreement except for the lectins WGA and ricin. The abundance of the terminal sialic acid structure bound by WGA increases in Table 4 while the sensitivity of LEC11to WGA decreases in Table 3. Perhaps the branch fucosylation in LEC11 cells disproportionately reduces the sensitivity of adjacent sialic acid groups to the WGA lectin. Neither of the two versions of the Ricin-binding substructure increase in Table 4 from the parent Pro¯5 cells while the sensitivity of LEC11 cells to ricin in Table 3 is 25 times higher. Perhaps the high level of SLex groups in LEC11 (Table 2) is a factor.

#### LEC12

Similarly to LEC11, the LEC12 mutant also exhibits expression of branch fucosylation activity, but due to activation of the Fut9 gene rather than Fut6 [47].Comparing the model-predicted enzyme activity results for LEC11 and LEC12 in Table 1, we see that the LEC12 prediction has even more branch fucosylation activity than LEC11, but does not include the increased a3SiaT activity compared to the Pro¯5 cells that LEC11 exhibits. Instead,LEC12 is predicted to have an increase in iGnT activity, which creates LacNAc repeats. Again, the predictions are consistent with compensating regulation for the loss in lactosamine. Or it could be an additional response of the cell line to the selective pressure of the lectins.

In accordance with the enzyme activity shifts, the model-predicted glycan features in Table 2 show much more branch fucose, Le^x^ and VIM-2 for LEC12 than for LEC11, and less SLe^x^. Lactosamine extensions are also greater for LEC12 than LEC11, but this change is not sufficient to maintain the unfucosylated lactosamine groups as high as either LEC11 or the parent Pro¯5.

The lectin sensitivities for LEC12 in Table 3 are more consistent with the lectin binding subgroup abundances for LEC12 in Table 4 than the corresponding comparison for LEC11 but the proper binding substructures for ricin are still questionable.

#### Lec13

Lec13 exhibits loss of *Gmds* gene expression, which is needed for synthesis of GDP-Fuc [48].The model-predicted enzyme activities in Table 1 show the expected decrease in a6FucT activity in the Lec13 mutant to only 3% of the level in CHO Pro¯5. In addition, the predicted activities of ManII and GnTII are about double the corresponding activities of the CHO Pro¯5, while GnTIV and GnTV are also somewhat increased (Table 1). Compared to the parent Pro¯5 cells, the predicted glycan features for Lec13 in Table 2 have less monoantennary and hybrid structures and more tetraantennary structures. This results in more lactosamine groups than the Pro¯5 cells. This could be another example of regulatory compensation, in this case providing increased lactosamine to compensate for the greatly reduced core fucose. Or it could be an additional response of the cell line to the selective pressure of the lectins.

For Lec13 the lectin sensitivities in Table 3 are quite consistent with the model-predicted binding substructure abundances in Table 4.

#### LEC30

LEC30 is related to LEC11 and LEC12 in that it also exhibits branch α-1,3 fucosylation, in particular producing VIM-2 and Lewis X structures. This has been shown to be due to activation of both the Fut4 and Fut9 genes [47].In addition to the expected high level of branch fucosylation activity (a3FucT), the model predicted significant shifts in other enzyme activities. ManII, GnTII and a6FucT are much higher than in the Pro¯5 cells while iGnT is much lower. GnTIV and GnTV activities are also significantly higher.

The predicted impacts of these shifts on the glycan features are shown in Table 2. Here we see much higher branch and core fucose, and much lower high mannose, hybrid and monoantennary structures, as well as fewer lactosamine extensions. Triantennary and tetraantennary structures are predicted to be significantly higher than in the parent Pro¯5 cells. Le^x^, SLe^x^ and VIM-2 structures are all elevated compared to the parent Pro¯5 cells.

To some extent the predicted shifts are consistent with compensatory regulation of the glycosylation enzymes. In this case the shift from high mannose, hybrid and monoantennary glycans to triantennary and tetraantennary glycans could add lactosamine groups to partially compensate for the loss of lactosamine groups through branch fucosylation. However the decreases in iGnT and SiaT, and the corresponding decrease in lactosamine extensions and sialic acid seem to go in the opposite direction.

Except for the same ricin binding site fucosylation issue as for LEC11 and LEC12, the lectin sensitivities for LEC30 in Table 3 are quite consistent with the model-predicted lectin binding substructure abundances in Table 4.

### Discussion

The glycosylation model makes two kinds of predictions from experimental data: the set of glycan structures that explain an experimental mass spectrum, and the relative enzyme activities that account for the glycan structures. The prediction of the glycan structures underlying a measured mass spectrum is fairly robust, since many mass spectral peaks result from unique structures, and the overall set of structures predicted by the model result from the actions of the enzymes included in the model. As long as a good fit to the mass spectrum is obtained the model-predicted set of glycan abundances is a reasonable quantification of the mass spectrum. The production of these structures also implies an overall extent of reaction associated with each enzyme.

The enzyme activities that explain these extents of reaction are less certain. In this study we are only concerned with how the relative enzyme activities shift from one case to another, not on the absolute values of the activities. Even so, the comparisons depend on the assumption that everything in the model except for the enzyme activities is fixed between the cases, and that the model-predicted activity shifts are relatively insensitive to the particular choices made for various model parameters. This is not always the case. For example, in the LEC10 analysis the enzyme activity shifts from the base Pro¯5 cells were calculated with different assumed distributions of the GnTIII enzyme, in each case holding the distribution the same for each of the two cell types. While the same directional results were obtained for each distribution, the magnitudes of the activity shifts were significantly higher for one assumed distribution than they were for another.

The glycosylation functions lost by the loss-of-function mutants have been well explained in terms of defects in genes that are necessary for those functions. However, the model-based approach has predicted enhancements to other glycosylation functions that were not expected from previous studies. For example, it seems that the loss in GnTV function for the Lec4 cell line was significantly compensated for by a model-predicted increase in GnTIV activity. Similar effects were observed in the other loss-of-function mutant cell lines. This could be due to unobserved genetic changes selected by the same lectin stresses that selected the known gene mutations for these cell lines, or it could be due to regulatory effects within the cells that tend to stabilize cellular functions. In the Lec4 case the effect is very similar to the response of a variety of cell lines to loss of ManII or GnTII, where the resulting loss of lactosamine-containing complex glycans was compensated for by a regulatory mechanism to increase production of lactosamine repeats on hybrid glycans [35]. The authors also showed that the regulation does not involve any changes in expression level of glycosylation enzymes or sugar-nucleotide donor synthesis but takes place through self-correction mechanisms in the Golgi apparatus itself that involve shifting of the glycosylation reactions between the Golgi compartments.

In two of the loss-of-function cell lines, Lec1 and Lec3.2.8.1, the persistence of complex glycans in cells without any GnTI activity has been attributed by North et al. to contamination of the samples with small amounts of bovine serum used in culturing the cells. Given the rather small amounts of complex glycan peaks found in the mass spectrum this is a reasonable explanation. However the shifts in glycan features resulting from increased enzyme activities in the other cell lines are much larger and cannot be explained by this source.

In the case of the gain-of-function mutants, enzymes that are inactive in the base Pro¯5 cells have become activated, and some of the specific activated genes have been identified. But the underlying mechanism of this activation is unknown in most of the cases. The model-based analysis predicts that other enzymes, which are already active in the base cells, have increased their activities in the mutant cell lines compared to the base cell line. Again this could be part of the evolutionary response of the cell lines to the lectin stresses or part of a regulatory compensation. In at least some of the cell lines, however, the enzyme activity shifts do not appear consistent with the maintenance of lactosamine level in the glycans.

An important goal of modeling glycosylation is to predict what kind of enzyme activity profile can be engineered to produce a desired change in glycan profile. To the extent that enzyme expression levels can be engineered without engendering unforeseen regulatory compensation, the current version of the model could be a useful tool for predicting the desired changes. Indeed essentially the same model has already been used by others to do just that. As discussed in the Introduction, an earlier version of the model has been used by McDonald et al.[21] to predict what changes in enzyme activities would be required to increase N-glycan branching. In addition to the obvious increases in GnTIV and GnTV enzyme activities, the model predicted that a decrease in galactosyltransferase activity would significantly increase the desired structures. The authors then glycoengineered a number of cell lines with various shifts in enzyme expression and verified these predictions experimentally. This clearly indicates that the model has predictive power.

The current study provides some verification of the model-based approach in that the predictions are consistent with previously published results on the subject cell lines. However, the further model predictions not previously published have yet to be verified.

Even better predictions could be made if the model included regulatory mechanisms that are internal to the Golgi apparatus. Given that enzyme or donor cosubstrate redistribution within the Golgi compartments may play a role in this regulation, it would be very useful to understand the physical processes involved in the enzyme localization and include an approximation of those processes in the model.

Jimenez del Val et al. have investigated an optimization approach to localizing the enzymes in their model, where the spatial distributions of the enzymes are adjusted to minimize the enzyme concentrations required to accomplish the glycosylation reactions [24]. However this approach could be problematic for certain enzymes. For example, as shown above in the LEC10 analysis above, locating the GnTIII enzyme earlier in the Golgi apparatus decreases the amount of GnTIII required in the model to match the data but increases the required amounts of a number of other enzymes. A more fundamental mechanistic approach would be desirable.

It is remarkable that the model-based approach is able to infer structural details from mass spectra that would not be expected to contain that information. For example, any member of one of the two types of triantennary glycans, where the third branch is linked to the Manα3 vs. the Manα6, will mirror a corresponding member of the other group with identical mass where the third branch is just switched to the other mannose group. Just looking at the spectrum one mass peak at a time gives no information about how much of each type is present. However, the addition of various moieties, such as a fourth branch or lactosamine repeat, affect subsequent reactions of other enzymes as discussed in the Methods and Models section. These differences cause different amounts of subsequent glycans to be produced, so fitting the model to all the mass spec data can potentially differentiate these pathways. This was clearly demonstrated in the case of the Lec4 cell line.

The results in this study were based on mass spectra with no other input data. But the model-based approach is not at all limited to use only this type of data. Any type of measurement that can be calculated from a complete set of glycan structures can be used simultaneously with any other such measurement to adjust the model enzyme activities to match the data. Alternatively, once the model has been adjusted to match a base case glycan profile, the system could be used to predict what shifts in multiple enzyme activities are needed to produce some desired shift in glycan features.

## Conclusions

Predictions have been made for the CHO-cell parent strain and nine mutant cell lines. The model-based analysis predicts shifts in glycosylation enzyme activities between the various cell lines that are consistent with known biochemical shifts for each of the cell lines. The model also predicts unexpected shifts in enzyme activities that may be associated with regulatory compensation or unknown genetic shifts in these cell lines. However, these latter shifts have yet to be verified. The analysis also produces completely automatic annotation of the mass spectral peaks.

## Methods and models

### Model framework

Glycan structures are expressed using a condensed version of IUPAC linear formulas [49] with some minor modifications. The first modification is to order the branches at a branch point based just on the branch locants (the carbon atom number on the monosaccharide to which each branch is attached) without regard to the lengths of the branches(branch length is used by the IUPAC scheme). In addition, the sugar abbreviations have been replaced with the shorter abbreviations of the Linear Code [50], but without using the branch ordering scheme of the Linear Code. Table 7 includes the sugar codes used in glycan structures for our model. For example, the initial 9 -mannose N-glycan is represented as "Ma2Ma2Ma3(Ma2Ma3(Ma2Ma6)Ma6)Mb4GNb4GN;Asn". This scheme provides linear formulas that are general, are easily readable by humans and are unique for each glycan structure. For graphical annotations we use the CFG recommended graphical notation for glycans. See http://www.functionalglycomics.org/static/consortium/CFGnomenclature.pdf for details.

**Table 7:** Reaction rules.

Index	Enzyme	Cosubstrate	Coproduct	Substrate	Product	Constraint
1	ManI	water	mannose	(Ma2Ma	(Ma	~*2Ma3(…Ma6)Ma6 & ~Ga3
2	ManI	water	mannose	(Ma3(Ma2Ma3(Ma6)Ma6)	(Ma3(Ma3(Ma6)Ma6)	~Ga3
5	ManII	water	mannose	(Ma3(Ma6)Ma6	(Ma6Ma6	(GNb2|Ma3 & ~Gnbis
6	ManII	water	mannose	(Ma6Ma6	(Ma6	(GNb2|Ma3 & ~Gnbis
7	a6FucT	GDP-Fuc	GDP	GNb4GN	GNb4(Fa6)GN	GNb2|Ma3 & #A = 0 & ~Gnbis
8	GnTI	UDP-GlcNAc	UDP	(Ma3(Ma3(Ma6)Ma6)Mb4	(GNb2Ma3(Ma3(Ma6)Ma6)Mb4	
9	GnTII	UDP-GlcNAc	UDP	(GNb2|Ma3(Ma6)Mb4	(GNb2|Ma3(GNb2Ma6)Mb4	
10	GnTIII	UDP-GlcNAc	UDP	GNb2|Ma3	GNb2|Ma3(GNb4)	~Ab & ~Gnbis & ~(Ma6Ma6)Mb4
11	GnTIV	UDP-GlcNAc	UDP	(GNb2Ma3	(GNb2(GNb4)Ma3	~Gnbis
12	GnTV	UDP-GlcNAc	UDP	(GNb2Ma6	(GNb2(GNb6)Ma6	~Gnbis
13	iGnT	UDP-GlcNAc	UDP	(Ab4GN	(GNb3Ab4GN	
14	b4GalT	UDP-Gal	UDP	(GN	(Ab4GN	~*GNb4)(…Ma6)Mb4
15	a3SiaT	CMP-NeuAc	CMP	(Ab4GN	(NNa3Ab4GN	
24	a3FucT	GDP-Fuc	GDP	(…Ab4GNb	(Fa3(…Ab4)GNb	
19	a3GalT	UDP-Gal	UDP	(Ab4GN	(Aa3Ab4GN	

The "Index" column is an arbitrary identifier for each rule. The "Cosubstrate" is a chemical compound that reacts with the substrate. The "Coproduct" is an additional product of the reaction. The "Substrate" string specifies the substructure within a substrate upon which the enzyme acts. The "Product" string shows how the enzyme changes the substructure. The "Constraint" string indicates additional requirements of the substrate structure for the enzyme to act. Symbol definitions: "A": galactose; "F": fucose; "G": glucose; "GN": N-acetylglucosamine; "M": mannose; "NN": N-acetylneuraminic acid (sialic acid); "a": alpha; "b": beta.

The model framework includes a computer program that generates a complete reaction network based on a list of starting structures, and a set of reaction rules for each enzyme (Table 7). Each reaction rule contains a substrate specification string, a product modification string, and a constraint string specifying additional structural constraints on the substrate that are required for the reaction to occur. These specification strings within each reaction rule utilize a set of abbreviations shown in Table 8. Each substrate specification string is applied to each structure currently in the structure list to determine which structures are substrates for each rule (see [28] and [29] for examples). For structures that satisfy the substrate specification for a reaction rule, the product structure is determined from the product specification string of the rule. If the product structure so obtained is not in the current structure list, it is added to the list. At the same time, the new reaction is added to a reaction list. The reaction list includes the enzyme, substrate, cosubstrate, product and coproduct strings for each reaction. The list of rules in Table 7 is sufficient to produce most of the N-glycans present in CHO cells. Structures containing sulfates and phosphates have not been included because these groups are lost in the sample preparation for mass spectrometry used in the analyzed data. These groups could easily be added to the model if some data on their abundance were available.

**Table 8:** Reaction rule abbreviations.

Symbol	Meaning	String expression
-	Single ligand (no branch)	Any string (possibly empty) with parentheses matched but no final ")"
…	Ligand(s)	Any string (possibly empty) with parentheses matched
_	Continuation toward root	Any string (possibly empty) where every "(" is matched with a following ")"
|	Possible branch point	Empty string or "(…)" or ")" or ")(…)"
*	Reaction site	Position of first difference between substrate string and product string
Gnbis	Bisecting GN	Ma3(GNb4)(…Ma6)Mb4

These abbreviations are used within substrate strings, product strings, rule constraints and adjustment rules to describe glycan substructures.

The model framework also includes a network pruning algorithm that heuristically estimates the abundances of the generated structures and drops reactions from the list that lead to negligible structures. It additionally incorporates a molecular mass cutoff to limit the size of the glycans generated to those masses included in the mass spectral data range. This process is repeated until no new reactions can be generated. Starting with 9-mannose glycans, and including an inert structure with an additional glucose residue (Ga3), the rules of Table 7 generate a pruned reaction network containing 19,413 structures and 50,605 reactions. The complete list of structures included in the model is given as Supporting Information S1 Table. The maximum mass cutoff used to generate this network was 5000 on a permethylated basis and network pruning was enabled. (To improve the matching with experimental data, a larger network, containing 34,872 structures and 100,464 reactions was used for the LEC30 cell line analysis. The structures used for LEC30 are shown as Supporting Information S2 Table.)

The equations of the model assume Michaelis-Menten reaction kinetics, with the product structure competing for the same enzyme site as the substrate structure, and the donor cosubstrate occupying a second site on the enzyme. For a substrate P_i_ reacting with a donor cosubstrate UDP-S to give a product P_i+1_ and a coproduct UDP driven by enzyme E the equation for the reaction rate *r* takes the form:

$$\begin{array}{ll} r & =\frac{k_{f}[{\text{E}}_{t}]\left(\left[\text{UDP-S}][{\text{P}}_{i}]-\frac{1}{K_{eq}^{\prime }}[{\text{P}}_{i+1}][\text{UDP}\right]\right)}{K_{mi}\left(K_{md}+[\text{UDP-S}]\right)\left(1+\sum_{j}^{}\frac{\left[{\text{P}}_{j}\right]}{K_{mj}}\right)}\\ K_{eq}^{\prime } & =\frac{k_{f}K_{m,i+1}}{k_{r}K_{md}K_{mi}}=\frac{{\left[{\text{P}}_{i+1}\right]}^{*}{\left[\text{UDP}\right]}^{*}}{{\left[{\text{P}}_{i}\right]}^{*}{\left[\text{UDP-S}\right]}^{*}} \end{array}$$

Here the brackets indicate the concentration of the corresponding compound. The summation in the denominator is over all the substrates that compete for the enzyme.

The values of the kinetic parameters for a given enzyme can vary significantly for different substrates. This was accommodated by selecting base values for these parameters for each reaction rule (Table 9) and adding a set of structure-dependent adjustment rules (Table 10). Each adjustment rule includes a condition on the substrate structure and multipliers to apply to each of the three parameters whenever the condition is satisfied. Development of these parameter values and adjustments for CHO and human cell enzymes are detailed in prior studies [4,28,29].

**Table 9:** Reaction rate parameters and enzyme references.

Index	Enzyme	kf	Km	Kmd	EC No.	Gene ID
1	ManI	1923.75	827	0	3.2.1.113	MAN1A1, MAN1A2, MAN1B1, MAN1C1
2	ManI	100	5000	0	3.2.1.113	MAN1A1, MAN1A2, MAN1B1, MAN1C1
5	ManII	1923.75	200	0	3.2.1.114	MAN2A1, MAN2A2
6	ManII	1923.75	100	0	3.2.1.114	MAN2A1, MAN2A2
7	a6FucT	253	25	46	2.4.1.68	FUT8
8	GnTI	990	260	170	2.4.1.101	MGAT1
9	GnTII	1320	190	960	2.4.1.143	MGAT2
10	GnTIII	607.2	190	3100	2.4.1.144	MGAT3
11	GnTIV	187	3400	8300	2.4.1.145	MGAT4A, MGAT4B
12	GnTV	1410	130	3500	2.4.1.155	MGAT5
13	iGnT	24.66	700	55	2.4.1.149	B3GNT1, B3GNT2
14	b4GalT	8712	150	0	2.4.1.38	B4GALT1, B4GALT2, B4GALT3
15	a3SiaT	484.1	260	57	2.4.99.6	ST3GAL3
24	a3FucT	25	1400	9	2.4.1.152	FUT4, FUT7, FUT9
19	a3GalT	190	1150	12600	2.4.1.87	GGTA1

Symbol definitions: kf forward rate coefficient (min^-1^); Km Michaelis-Menten constant for substrate (μM); Kmd Michaelis-Menten constant for donor cosubstrate (μM). "Index refers to the rule number in Table 5. Values of Kmd equal to zero in the table imply that the enzyme is always saturated with the donor cosubstrate (Kmd/[Donor] ~0). This was used as a default value when no measured values were available.

**Table 10:** Adjustment rules.

Index	Rule	kf	Km	Kmd
1	#M = 9	1	6.552	1
1	#M = 8	1	4.024	1
1	#M = 7	1	1.696	1
1	Ma2Ma3(…Ma6)Ma6	1	8.620	1
10	~GNb2|Ma6	1	20.000	1
11	~GNb2|Ma6	1	5.000	1
11	Ab4GNb2|Ma6 or Ab4GNb6)Ma6	1	1.500	1
11	GNb6)Ma6	1	0.178	1
12	GNb4)Ma3	1	0.692	1
13	*_Ma3	1	10.000	1
13	*_GNb2Ma6	1	4.000	1
13	*_GNb2Ma3	1	4.000	1
14	*_GNb6)Ma6	1	0.800	1
14	*_GNb2|Ma6	1	5.400	1
14	*_GNb4)Ma3	1	0.667	1
14	Gnbis & GNb2|Ma6	1	3.620	1
14	~GNb2|Ma6	1	26.667	1
14	Ab4	1	0.343	1
15	#NN>1	1	5.000	1
24	(*Fa2Ab4	4.08	0.500	1

The "Adjust" column specifies an arbitrary identifier for each adjustment rule. The "Index" column specifies which reaction rule in Table 5 the adjustment applies to. The codes in the "Rule" column specify conditions on the substrate structure for the adjustment to apply. The "kf", "Km" and "Kmd" columns are multipliers applied to the corresponding reaction rate parameters in Table 9.

Other parameters needed for the calculations, include compartment residence times, enzyme distributions between compartments, and donor cosubstrate concentrations shown in Table 11. Total glycan concentration was assumed to be 500 μM. These were estimated based on a variety of literature sources as detailed in prior studies [4,28]. The spatial distributions of enzymes were derived from beta distributions adjusted to best match the experimental mass spectra as shown in Table 5 and Fig 4. The assumed values are shown in Table 11.

**Table 11:** Golgi compartment parameters used in the model.

Compartment	1	2	3	4
Residence time, min.	5.556	5.556	5.556	5.556
Enzyme distributions				
ManI	0.340	0.453	0.188	0.019
ManII	0.126	0.419	0.371	0.085
a6FucT	0.126	0.419	0.371	0.085
GnTI	0.126	0.419	0.371	0.085
GnTII	0.126	0.419	0.371	0.085
GnTIII	0.002	0.041	0.244	0.713
GnTIV	0.126	0.419	0.371	0.085
GnTV	0.126	0.419	0.371	0.085
iGnT	0.126	0.419	0.371	0.085
b4GalT	0.002	0.041	0.244	0.713
a3SiaT	0.002	0.041	0.244	0.713
a3FucT	0.002	0.041	0.244	0.713
a3GalT	0.002	0.041	0.244	0.713
Donor concentrations				
water, M	56	56	56	56
GDP-Fuc, μM	5000	5000	5000	5000
UDP-GlcNAc, μM	9143	9143	9143	9143
UDP-Gal, μM	3810	3810	3810	3810
CMP-NeuAc, μM	2286	2286	2286	2286

As explained in the Results and discussion the spatial distributions of the enzymes was derived from beta distributions adjusted to best match the mass spectral data, as shown in Table 3 and Fig 4.

The Golgi stack is modeled as a set of four well-mixed compartments. Based on these assumptions, equations were derived to solve for the concentrations of each of the individual glycan structures in each of the Golgi compartments. Robust solution methods were devised to allow simultaneous solution of the approximately 80,000 nonlinear equations (140,000 for the LEC30 case) for the concentration of each of the glycan structures in each of the four compartments of the model. In addition to solving the model for a given set of model parameters, provision was also made to adjust parameters to match a given set of data. This was done using the Marquardt-Levenberg method with analytically calculated derivatives [51].

To map model glycan structure distributions to MALDI-TOF mass spectrometric data, a synthetic mass spectrum was obtained from the model-calculated glycan abundances. Details of the synthetic mass spectra calculations are given in [28.29]. Automatic parameter adjustment is used to bring the synthetic mass spectrum obtained from the model into agreement with the experimental mass spectra by adjusting enzyme activities for the different cell types.

Methods for experimental mass spectra processing have been enhanced from those methods described previously [28]. The same methods were used for baseline correction and peak integration, but a smoothing step was added between these steps to reduce the effect of noise on the peak maximum detection. A step was also added to eliminate isolated peaks that are not part of any isotopic satellite group of peaks. Such peaks are artifacts of instrument noise.

After processing, the experimental mass spectra still contain a significant number of minor peaks (actually isotopic satellite groups of peaks), which do not correspond to any glycans in the model. In most cases they do not correspond to any known N-glycans. Presumably these are artifacts of sample processing, or perhaps fragments produced in the mass spectrometer. In any event, to avoid confounding of the model parameter adjustment step, the preprocessed experimental spectra were further adjusted by projecting them onto only the masses contained in the model by means of a nonnegative linear regression method. This allows us to match the model parameters to only that part of the experimental mass spectrum explained by the model. However, in visually comparing the model spectrum to the experimental, for example in Figs 2 and 3, the original unprojected experimental spectra have been used.

### Experimental data

Experimental glycan structure measurements via MALDI-TOF mass spectrometry are available at the Consortium of Functional Glycomics (CFG) website which contains such results for a variety of cell lines and species.

The URLs for the particular data files used in this paper are listed in Table 12:

**Table 12:** Links to mass spectral data.

Cell line	URL
Pro¯5	http://functionalglycomics.org/coreCStatic/allmsdfiles/7950.msd
Lec1	http://functionalglycomics.org/coreCStatic/allmsdfiles/10388.msd
Lec2	http://functionalglycomics.org/coreCStatic/allmsdfiles/10392.msd
Lec3.2.8.1	http://functionalglycomics.org/coreCStatic/allmsdfiles/LEC3.2.8.1.msd
Lec4	http://functionalglycomics.org/coreCStatic/allmsdfiles/7654.msd
LEC10	http://functionalglycomics.org/coreCStatic/allmsdfiles/7656.msd
LEC11	http://functionalglycomics.org/coreCStatic/allmsdfiles/8433.msd
LEC12	http://functionalglycomics.org/coreCStatic/allmsdfiles/8437.msd
Lec13	http://functionalglycomics.org/coreCStatic/allmsdfiles/7658.msd
LEC30	http://functionalglycomics.org/coreCStatic/allmsdfiles/8449.msd

These cell types, with N-glycan mass spectra provided by Pamela Stanley's group, represent Lec1 (CHO Pro-Lec1.3C [41]), Lec2 (CHO Pro-Lec2.6A [42]), Lec3.2.8.1 (CHO Pro-Lec3.2.8.1.3B [52]), Lec4 (CHO Pro-Lec4.7B [44]),LEC10 (CHO Pro-LEC10.3C [45]), LEC11 (CHO Pro-LEC11.E7 [46]), LEC12 (CHO Pro-LEC12.1B [47]), Lec13 (CHO Pro-Lec13.6A [48,53]), LEC30 (CHO Pro-LEC30.H2 [47]). All are referred to in their short form for readability.

## Supporting information

S1 Fig Measured and calculated mass spectra. Complete annotated mass spectra for the 10 cell lines included in the paper in the same format used in Fig 2.
(PDF)

S1 Table List of components utilized for all models except LEC30. (CSV)

S2 Table List of components utilized for LEC30. (CSV)
