# Supplementary material for: Model-based analysis of N-glycosylation in Chinese hamster ovary cells
Source: PLoS One. 2017 May 9;12(5):e0175376. doi: 10.1371/journal.pone.0175376 (PMC5423595; doi:10.1371/journal.pone.0175376)
Supplement: S1 Fig — Complete annotated mass spectra for the 10 cell lines included in the paper in the same format used in Fig 2. (PDF) [file pone.0175376.s001.pdf]

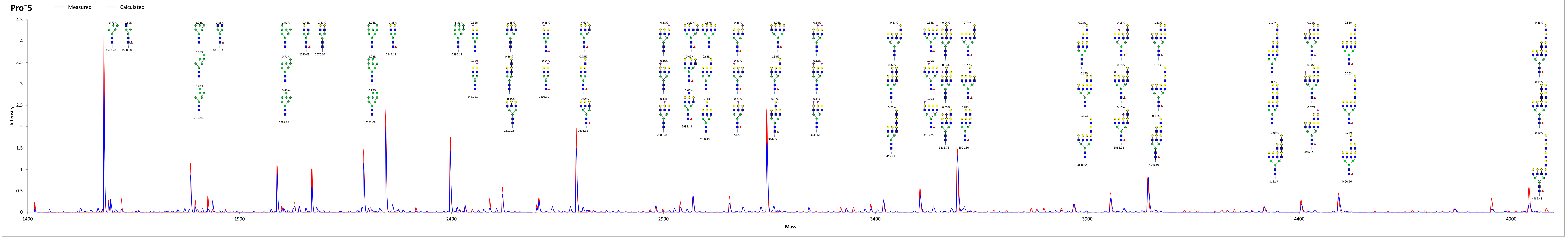
Pro¯5
Measured
Calculated
Intensity
Mass
4.5
4
3.5
3
2.5
2
1.5
1
0.5
0
1400
1900
2400
2900
3400
3900
4400
4900
1579.78
1590.80
1783.88
1835.93
1987.98
2040.03
2070.04
2192.08
2244.13
2396.18
2431.21
2519.26
2605.30
2693.35
2880.44
2938.48
2968.49
3054.52
3142.58
3241.61
3417.71
3503.75
3531.76
3591.80
3866.94
3952.98
4041.03
4316.17
4402.20
4490.26
4939.48

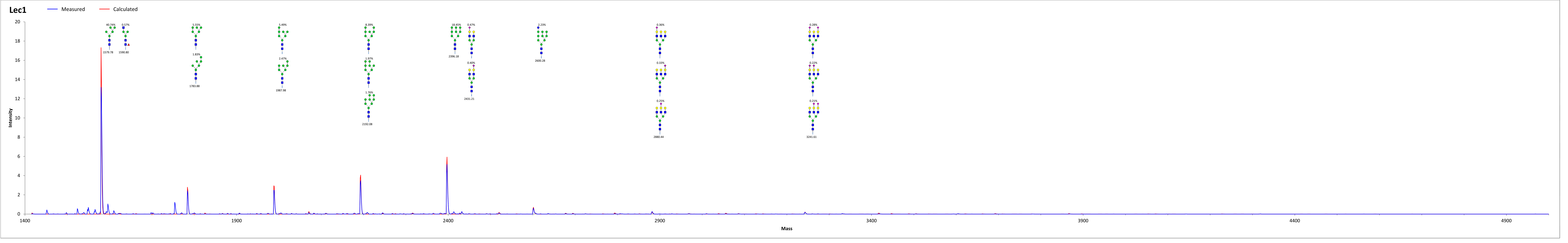
Lec1
Measured
Calculated
Intensity
Mass
20
18
16
14
12
10
8
6
4
2
0
1400
1900
2400
2900
3400
3900
4400
4900
40.74%
0.57%
1579.78
1590.80
5.55%
1.83%
1783.88
5.49%
2.47%
1987.98
8.39%
1.97%
1.76%
2192.08
18.45%
2396.18
0.47%
0.40%
2431.21
2.23%
2600.28
0.36%
0.33%
0.25%
2880.44
0.28%
0.22%
0.21%
3241.61

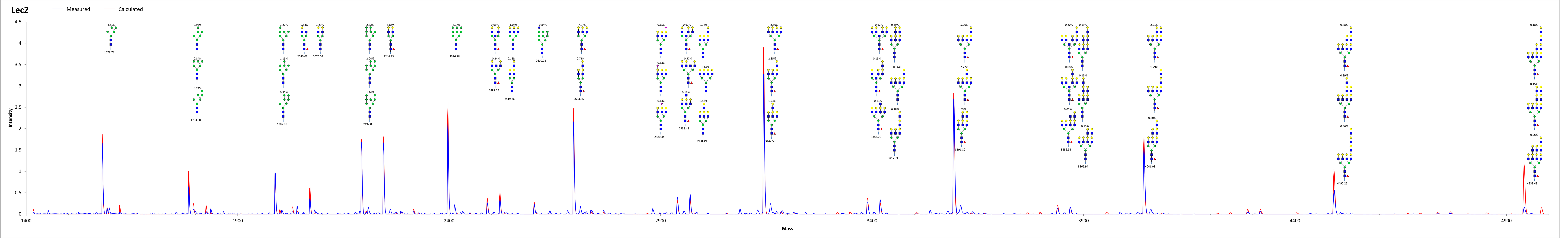
Lec2
Measured
Calculated
Intensity
Mass
4.5
4
3.5
3
2.5
2
1.5
1
0.5
0
1400
1900
2400
2900
3400
3900
4400
4900

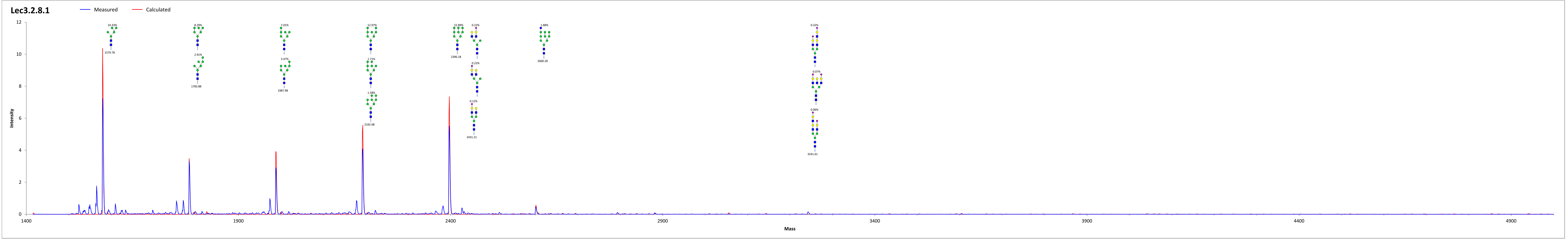

Lec3.2.8.1
Measured
Calculated
Intensity
12
10
8
6
4
2
0
24.33%
1579.78
8.29%
2.91%
1783.88
7.01%
3.47%
1987.98
12.97%
1.71%
1.58%
2192.08
22.89%
0.22%
2396.18
0.22%
0.12%
2431.21
1.88%
2600.28
0.32%
0.07%
0.06%
3241.61
1400
1900
2400
2900
3400
3900
4400
4900
Mass

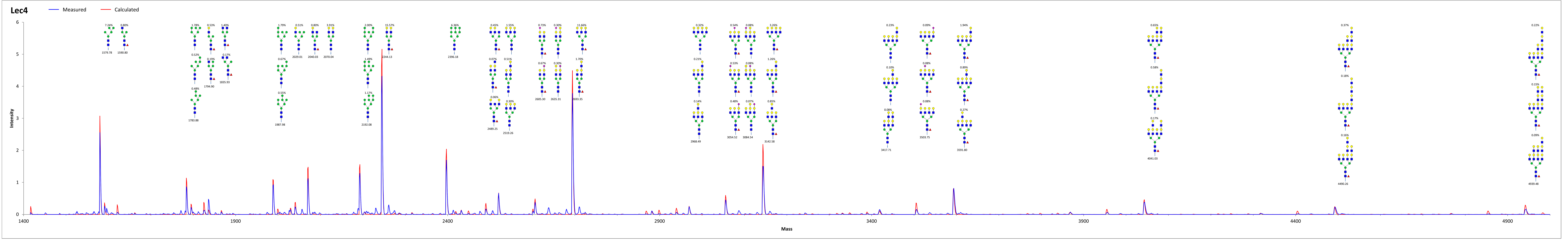

Lec4
Measured
Calculated
Intensity
Mass
0
1
2
3
4
5
6
1400
1900
2400
2900
3400
3900
4400
4900
1579.78
1590.80
1783.88
1794.90
1835.93
1987.98
2029.01
2040.03
2070.04
2192.08
2244.13
2396.18
2489.25
2519.26
2605.30
2635.31
2693.35
2968.49
3054.52
3084.54
3142.58
3417.71
3503.75
3591.80
4041.03
4490.26
4939.48

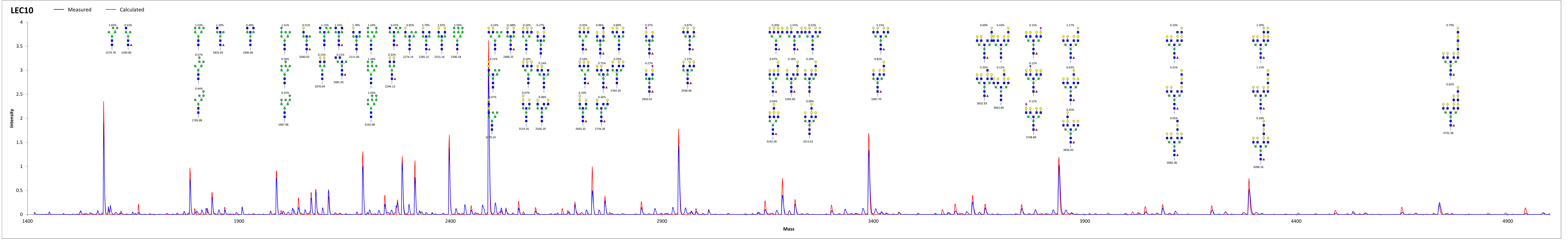
LEC10
Measured
Calculated
Intensity
Mass
1400
1900
2400
2900
3400
3900
4400
4900

LEC11

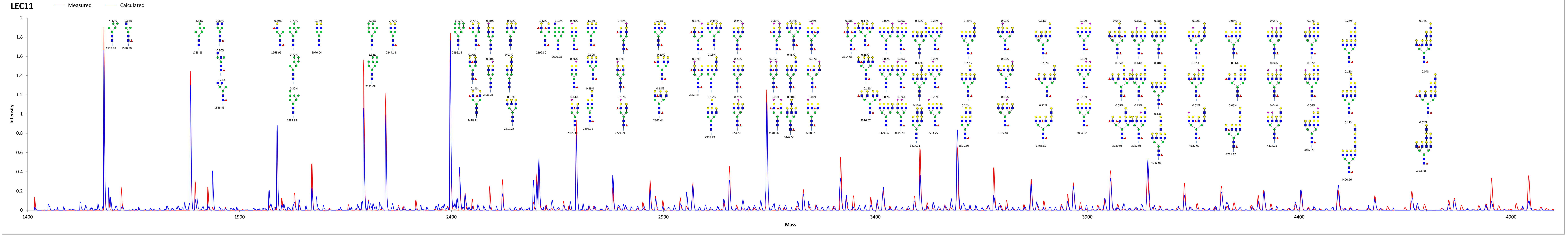

Measured
Calculated
Intensity
Mass
1400
1900
2400
2900
3400
3900
4400
4900
0
0.2
0.4
0.6
0.8
1
1.2
1.4
1.6
1.8
2

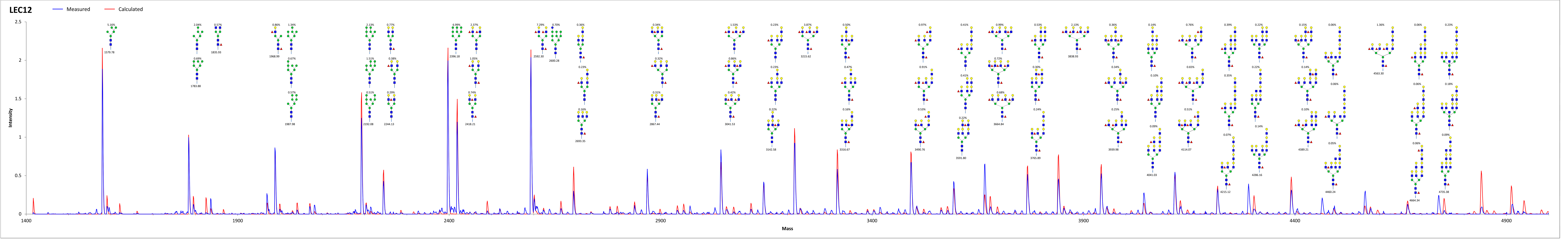
LEC12
Measured
Calculated
Intensity
Mass

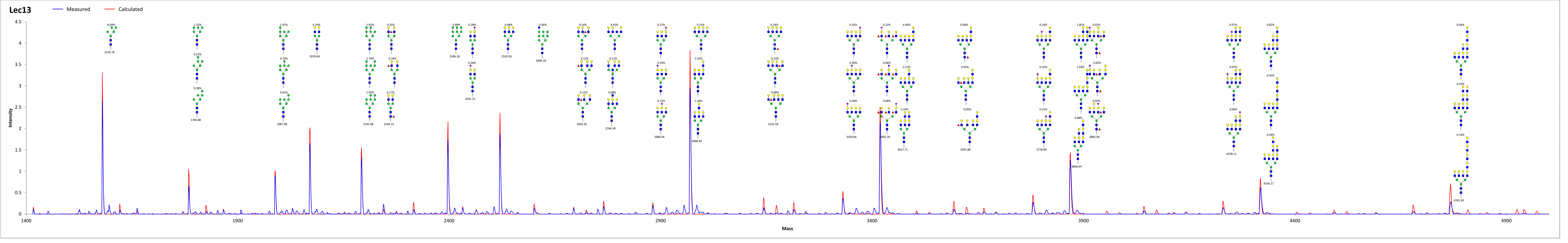

Lec13
Measured
Calculated
Intensity
Mass
4.5
4
3.5
3
2.5
2
1.5
1
0.5
0
1400
1900
2400
2900
3400
3900
4400
4900
1579.78
1783.88
1987.98
2070.04
2192.08
2244.13
2396.18
2431.21
2519.26
2600.28
2693.35
2764.39
2880.44
2968.49
3142.58
3329.66
3402.70
3417.71
3595.80
3778.89
3866.94
3881.94
4228.13
4316.17
4765.39

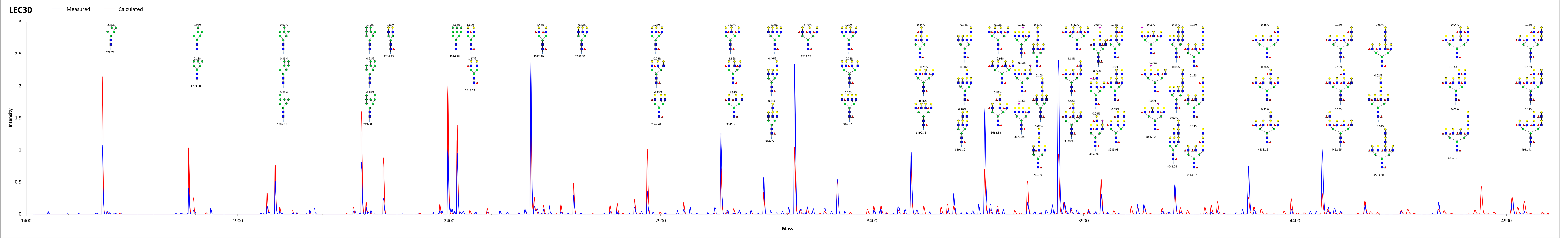

LEC30
Measured
Calculated
Intensity
Mass
3
2.5
2
1.5
1
0.5
0
1400
1900
2400
2900
3400
3900
4400
4900
1579.78
1783.88
1987.98
2192.08
2244.13
2396.18
2418.21
2592.30
2693.35
2867.44
3041.53
3142.58
3215.62
3316.67
3490.76
3591.80
3664.84
3677.84
3765.89
3838.93
3851.93
3939.98
4026.02
4041.03
4114.07
4288.16
4462.25
4563.30
4737.39
4911.48
